# Genome-wide analysis of sex-specific differences in the mother–child PELAGIE cohort exposed to organophosphate metabolites

**DOI:** 10.1038/s41598-023-35113-8

**Published:** 2023-05-17

**Authors:** Martina Capriati, Chunxiang Hao, Shereen Cynthia D’Cruz, Christine Monfort, Cecile Chevrier, Charline Warembourg, Fatima Smagulova

**Affiliations:** 1 Univ. Rennes, EHESP, Inserm, Irset (Institut de Recherche en Santé, Environnement et Travail) - UMR_S 1085, 35000 Rennes, France; 2 School of Medicine, Linyi University, Linyi, 276000 China

**Keywords:** Environmental impact, Embryonic induction

## Abstract

In recent decades, the detrimental effects of environmental contaminants on human health have become a serious public concern. Organophosphate (OP) pesticides are widely used in agriculture, and the negative impacts of OP and its metabolites on human health have been demonstrated. We hypothesized that exposure to OPs during pregnancy could impose damaging effects on the fetus by affecting various processes. We analyzed sex-specific epigenetic responses in the placenta samples obtained from the mother–child PELAGIE cohort. We assayed the telomere length and mitochondrial copy numbers using genomic DNA. We analyzed H3K4me3 by using chromatin immunoprecipitation followed by qPCR (ChIP‒qPCR) and high-throughput sequencing (ChIP-seq). The human study was confirmed with mouse placenta tissue analysis. Our study revealed a higher susceptibility of male placentas to OP exposure. Specifically, we observed telomere length shortening and an increase in γH2AX levels, a DNA damage marker. We detected lower histone H3K9me3 occupancy at telomeres in diethylphosphate (DE)-exposed male placentas than in nonexposed placentas. We found an increase in H3K4me3 occupancy at the promoters of thyroid hormone receptor alpha (THRA), 8-oxoguanine DNA glycosylase (OGG1) and insulin-like growth factor (IGF2) in DE-exposed female placentas. H3K4me3 occupancy at PPARG was increased in both male and female placentas exposed to dimethylphosphate (DM). The genome-wide sequencing of selected samples revealed sex-specific differences induced by DE exposure. Specifically, we found alterations in H3K4me3 in genes related to the immune system in female placenta samples. In DE-exposed male placentas, a decrease in H3K4me3 occupancy at development-related, collagen and angiogenesis-related genes was observed. Finally, we observed a high number of NANOG and PRDM6 binding sites in regions with altered histone occupancy, suggesting that the effects were possibly mediated via these factors. Our data suggest that in utero exposure to organophosphate metabolites affects normal placental development and could potentially impact late childhood.

## Introduction

A growing body of evidence indicates that exposure to environmental factors can have profound consequences on human health. The most detrimental effects occur during embryonic exposure due to global epigenetic reprogramming, high proliferation rates, and organogenesis events. According to Developmental Origin of Human Disease theory (DOHAD), formulated by David Barker, environmental conditions experienced in the first phases of development can have long-term effects on later phases of life^1^. This phenomenon is linked to the biological plasticity of development, which allows easy reprogramming of physiological functions in response to different stimuli. Consequently, in utero exposure to environmental pollutants can increase predisposition to different pathologies that can occur both in early and later phases of life.

In this study, we analyzed the effects of metabolites of organophosphate (OP) pesticides on the human placenta under the framework of the Human Biomonitoring for Europe Initiative (HBM4EU) to reveal new biomarkers of exposure to organophosphate pesticides. The general products of OP detoxification are dialkyl phosphates (DAPs), such as diethylphosphate (DE) and dimethylphosphate (DM), and these metabolites are normally detected in urine by mass spectrometry^2^. Studies have shown that exposure to OPs can damage mitochondrial functions due to the induction of oxidative stress^3^. OPs can induce hormone-related cancers^4^ and can affect the development of the nervous system^5^. In children, in utero exposure to OPs has been shown to be associated with a wide range of developmental pathologies, such as cognitive dysfunctions and lower IQ in boys^6^ and deficits in the Working Memory Index^7^. The consequences of early-life exposure to toxic substances could be studied by analyzing certain biomarkers in noninvasive human biological samples, such as umbilical cord blood and placenta. The placenta is a transient organ that serves as an exchange platform between the fetal and maternal circulation, and it is available in large quantities. The placenta develops from the trophectoderm (TE) at ~ 5 days postfertilization, which corresponds to the blastocyst stage in the preimplantation embryo. After implantation, the trophoblast cells start to invade the uterus with vascular remodeling of the mother’s blood vessels. Defects in trophoblast cell differentiation could induce pregnancy-related complications such as preeclampsia and could cause low birth weight in infants^8^.

It has been shown that transcription factors are important for early TE-specific gene expression, including *GATA3*^9^, *TEAD4*^10^, *TCFAP2C*^11^ and *EOMES*^12^. Studies have shown that many placental factors could be affected by environmental stress. For example, vascular endothelial growth factor A (*VEGFA*) expression was higher in the placenta of women who smoked, suggesting its role in increasing the risk of developing smoking-associated preeclampsia^13^. In utero exposure to cadmium, BPA and polychlorinated bisphenols has been reported to increase the expression of *KISS1* by three times, suggesting effects on steroid hormone pathways^14^. Human cytotrophoblast cells isolated from fresh placentas and exposed to BPA for 24 h showed proliferation inhibition and increased apoptosis, and these events were accompanied by a significant increase in tumor-necrosis factor alpha (*TNFA*) gene expression and protein levels^15^. Thus, environmental toxicants could induce alterations in important processes by modifying gene expression related to placental development and functions.

It is suggested that epigenetic mechanisms are extremely vulnerable during development due to the highly dynamic nature of the chromatin remodeling processes during development. Epigenetic mechanisms are essential not only for correct cell type-specific gene expression but also for safeguarding cell identity. Of the epigenetic mechanisms, DNA methylation is the most studied. Goodrich et al. showed that early-life exposure to lead, bisphenol A and phthalate metabolites led to hypomethylation of LINE1 and hypermethylation of the imprinted gene *IGF2* in the exposed group^16^. BPA exposure lowered CpG methylation of gene promoters associated with metabolic and oxidative stress^17^.

We hypothesize that alterations in epigenetic regulation can affect normal placental processes. These changes in epigenetic marks could affect the expression of genes that are critical for fetal development and growth. In this study, we explored the effects of exposure to OP metabolites (DE and DM) on human placenta from the mother–child PELAGIE cohort from Brittany, France. We focused mainly on DE exposure because we did not observe many statistically significant associations with exposure to DM.

We show that in utero exposure to OP leads to global alterations in important regulatory histones both in male and female placentas. Several consequences of these alterations are discussed. Our data suggest that in utero exposure to organophosphate metabolites affects normal placental development and could potentially impact late childhood.

## Results

### Telomere length, mtDNA copy number and γH2AX level analysis in the human placenta

A graphical overview of the experiments performed in this study is provided in Fig. S1. Telomere length and mitochondrial copy numbers are often used as markers of toxicological exposure. Telomeres are hexanucleotide repeats located at both ends of each chromosome surrounded by RNA and protein complexes. Telomere length is reduced with each cell division; thus, telomere length is a marker of cellular senescence^18^. Since telomeres could be damaged due to oxidative stress^19^, we chose this marker to assess the toxic effect of OP.

MtDNA is another common marker used in toxicological studies, as oxidative stress induced by toxicants could damage mitochondria and reduce their numbers. MtDNA is extracellular DNA, in contrast to the nuclear genome, which contains only two copies per cell, whereas the mitochondrial genome is present in multiple copies per cell (from 100 to 10,000), depending on the cell type^20^.

**Figure 1:**
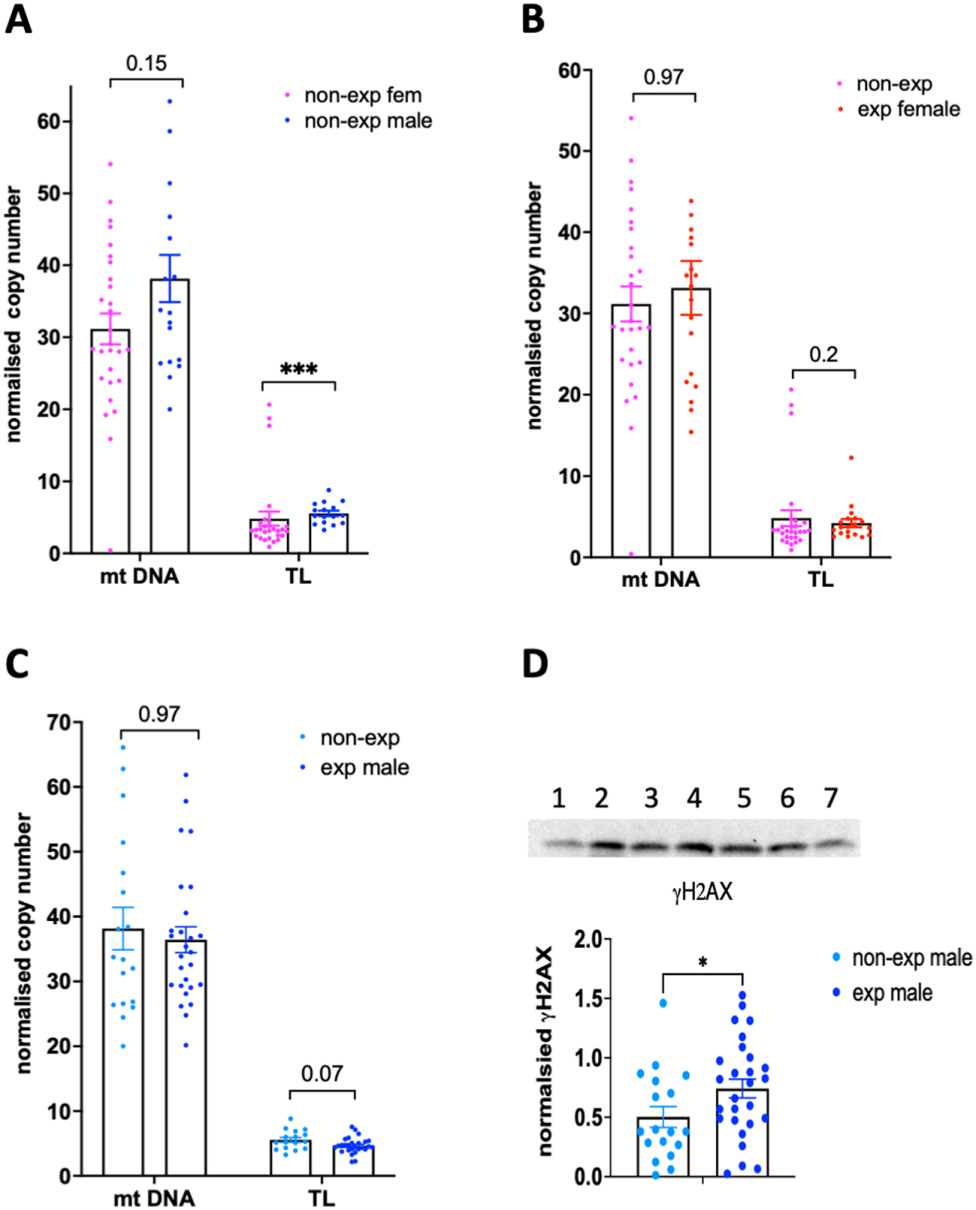
Copy number variations identified in human male and female placentas. (**A**) There was a tendency toward a higher number of mtDNA copies and longer telomeres in the nonexposed male placenta than in the nonexposed female placenta. (**B**) Exposure to DE does not significantly affect the mtDNA or TL copies in female placentas. (**C**) There is a tendency toward a decrease in TL in DE-exposed male placentas. (**D**) Quantitative analysis of gH2AX in the male group; top, representative image of WB analysis of placental samples; bottom, quantitative analysis of gH2AX. Equal amounts of DNA were taken for each reaction. The number of copies was normalized to the *RPLP0* gene. *p < 0.05, ***p < 0.001, Mann‒Whitney test.

We performed an analysis using genomic DNA and normalized the mtDNA copies and TL to the *RPLP0* gene. The analysis showed that the mtDNA copy number was 1.2 times higher in nonexposed males than in nonexposed females; however, the difference was not significant (p value = 0.15) (Fig. 1A). In addition, the analysis showed that TL was longer in males than in females (p < 0.05, Fig. 1A).

We also compared differences in mtDNA and TL between the DE and nonexposed groups in both sexes: in response to DE, we did not observe changes in mtDNA or TL copies in females (Fig. 1B). MtDNA copies were not affected, but TL in males was lowered by 16% (p = 0.07) (Fig. 1C).

Since alterations in TL could be a marker of toxicity, which very often induces DNA damage, we assessed whether DE exposure could induce DNA damage. The phosphorylation of the histone variant H2AX on the Ser-139 residue, generating ɣH2AX, is a rapid cellular response to double strand breaks and it is a key step to initiate the DNA damage response^21^. To this end, we extracted histones from male placentas and performed quantitative analyses of γH2AX levels using WB. The quantitative analysis showed that there was a significant increase (1.5 times) in γH2AX in DE-exposed male placentas compared to nonexposed placentas, suggesting a genotoxic effect of DE on human placentas (Figs. 1D, S2).

To examine other physiological parameters that may contribute to low resistance to negative factors, we considered maternal age, body mass index and smoking status. We found that TL and ɣH2AX remained statistically significant after controlling for maternal age, body mass index and smoking status (Table 1).

**Table 1:** Adjusted changes in TL and ɣH2AX levels male placentas in the group exposed to DE compared to the nonexposed group considering maternal age, body mass index, and smoking status.

	Sex	Mean [95% CI]^a^	p value
TL	Male	− 0.93 [− 1.72; − 0.14]	0.022
ɣH2AX	Male	0.24 [0.00; 0.48]	0.048

^a^Beta coefficients and 95% confidence intervals obtained from multivariable regression models.

Thus, the analysis of telomere length and gH2AX suggests that DE imposes damaging effects on human male placentas.

### Reduced histone H3K9me3 levels at male placental telomeres

**Figure 2:**
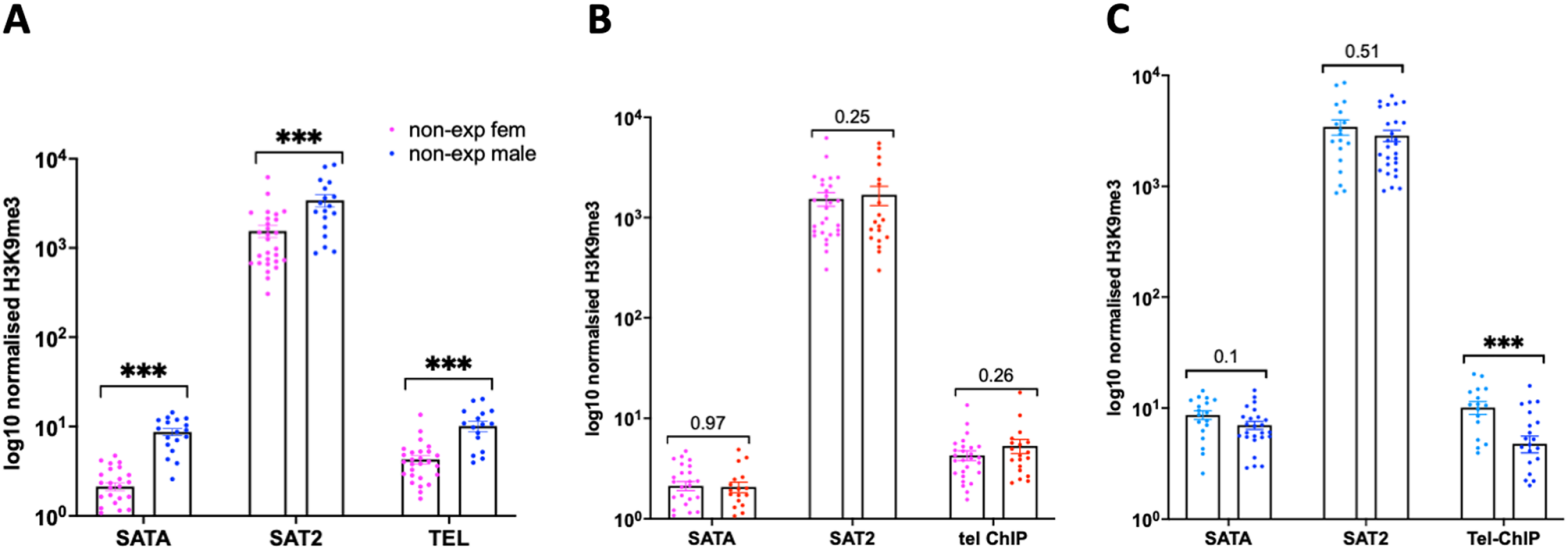
Histone H3K9me3 occupancy at the repetitive regions of the genome. (**A**) Analysis of H3K9me3 showed higher occupancy of H3K9me3 at satellites and telomeres in nonexposed male placentas than in nonexposed female placentas. (**B**) There were no significant changes in H3K9me3 levels in female placentas exposed to DE. (**C**) In exposed males, there was a tendency toward a decrease in SATA and a decrease in H3K9me3 at telomeres, ***p < 0.001, Mann‒Whitney test.

H3K9me3 is an important silencing regulatory mark, and it is enriched at telomeres and centromeres and repeat-containing regions. Deregulation of this mark was previously observed following exposure to the herbicide atrazine^22^. Since an alteration in H3K9me3 at telomeres has been shown to contribute to telomere instability^23^, we decided to address whether the changes in telomere length could be associated with H3K9me3 changes. To this end, we performed ChIP‒qPCR and analyzed the H3K9me3 occupancy at a few elements in centromeres (SATA), pericentromeres (SAT2) and telomere regions (Tel). Our quantitative analysis revealed a profound difference in H3K9me3 levels at the centromeres, pericentromeres and telomeres between nonexposed males and nonexposed females (Fig. 2A). We did not observe any changes in H3K9me3 in the analyzed targets in female placentas (Fig. 2B). Histone H3K9me3 did not show significant changes in SAT2 targets, but it tended to decrease in centromere SATA (p = 0.1) in DE-exposed male placentas (Fig. 2C). In contrast to females, we detected a significant decrease in H3K9me3 occupancy at telomeres (FC = 2.1, p < 0.001) in DE-exposed male placentas, suggesting a possible impact of DE on important regulating histone marks (Fig. 2C).

### Histone H3K4me3 is altered at *IGF2*, *THRA* and *OGG1* in the female placenta

To reveal the effects of exposure on the regulation of important developmental genes, we analyzed histone H3K4me3 occupancies at several targets, including an imprinted gene (*IGF2*), thyroid receptors (*THRA* and *THRB*), kisspeptin (KISS1) and a marker of DNA damage, namely, 8-oxoguanine DNA glycosylase (*OGG1*).

**Figure 3:**
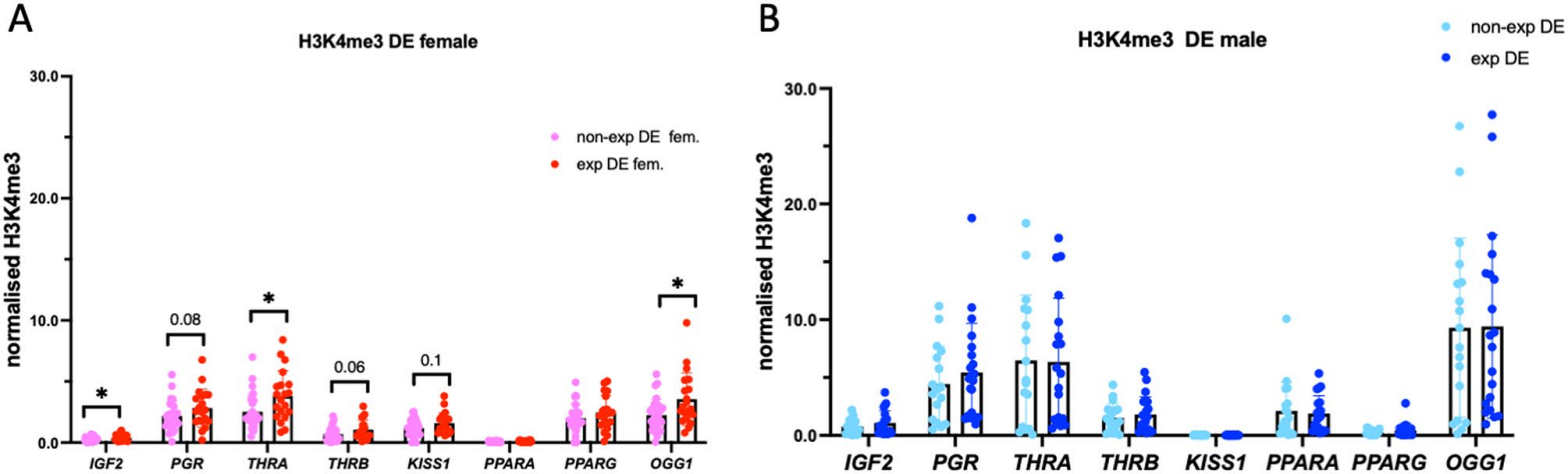
Histone H3K4me3 occupancy at developmental genes in DE placentas. The effects of DE on female (**A**) or (**B**) male placentas, *p < 0.05, **p < 0.01, Mann‒Whitney test.

Our analysis revealed that placentas exposed to DE have increased H3K4me3 at the *IGF2, THRA* and *THRB* promoters by 1.7-, 1.5- and 1.6-fold, respectively (Fig. 3A). We observed that the female placenta showed a 1.6-fold increase in promoter occupancy of the DNA repair factor *OGG1* (Fig. 3A). Male placentas did not show significant differences in H3K4me3 at the studied targets (Fig. 3B).

Similarly, we reanalyzed our data by comparing our data with other physiological parameters, such as maternal age, body mass index and smoking status. We found that alterations in *IGF2*, *OGG1* and *THRA* remained statistically significant in female placentas. In addition, we observed a significant change in *IGF2* in the male placenta after controlling for maternal age, body mass index and smoking status (Table 2).

**Table 2:** Adjusted changes in placentas in the group exposed to DE compared to the nonexposed group considering for maternal age, body mass index, and smoking status.

	Sex	Crude analyses^a^		Adjusted analyses^b^	
		Mean [95% CI]^a^	p value	Mean [95% CI]^a^	p value
IGF2	Female	0.17 [0.04; 0.30]	0.013	0.15 [0.01; 0.29]	0.031
OGG1	Female	1.35 [0.31; 2.41]	0.013	1.22 [0.16; 2.29]	0.025
THRA	Female	1.29 [0.24; 2.34]	0.017	1.15 [0.08; 2.22]	0.036
THRB	Female	0.41 [− 0.00; 0.81]	0.050	0.36 [− 0.06; 0.77]	0.095

^a^Beta coefficients and 95% confidence intervals obtained from crude regression models.

^b^Beta coefficients and 95% confidence intervals obtained from multivariable regression models adjusted for maternal age, body mass index, and smoking status.

We also analyzed H3K4me3 in the context of DM exposure. Our analysis revealed that placentas exposed to DM had increased H3K4me3 at the *PPARG* promoter by 1.8- and 2.8-fold in female placentas (Fig. S3A) and male placentas, respectively (Fig. S3B). H3K4me3 occupancy at *OGG1* showed a tendency to increase (p = 0.06 for both) by 1.4- and 1.6-fold in female and male placentas, respectively.

Thus, our H3K4me promotor occupancy analysis showed that exposure to DE and DM causes alterations in genes encoding imprinting, thyroid hormone receptor and DNA repair factors.

### Histone H3K4me3 by ChIP-seq analysis

**Figure 4:**
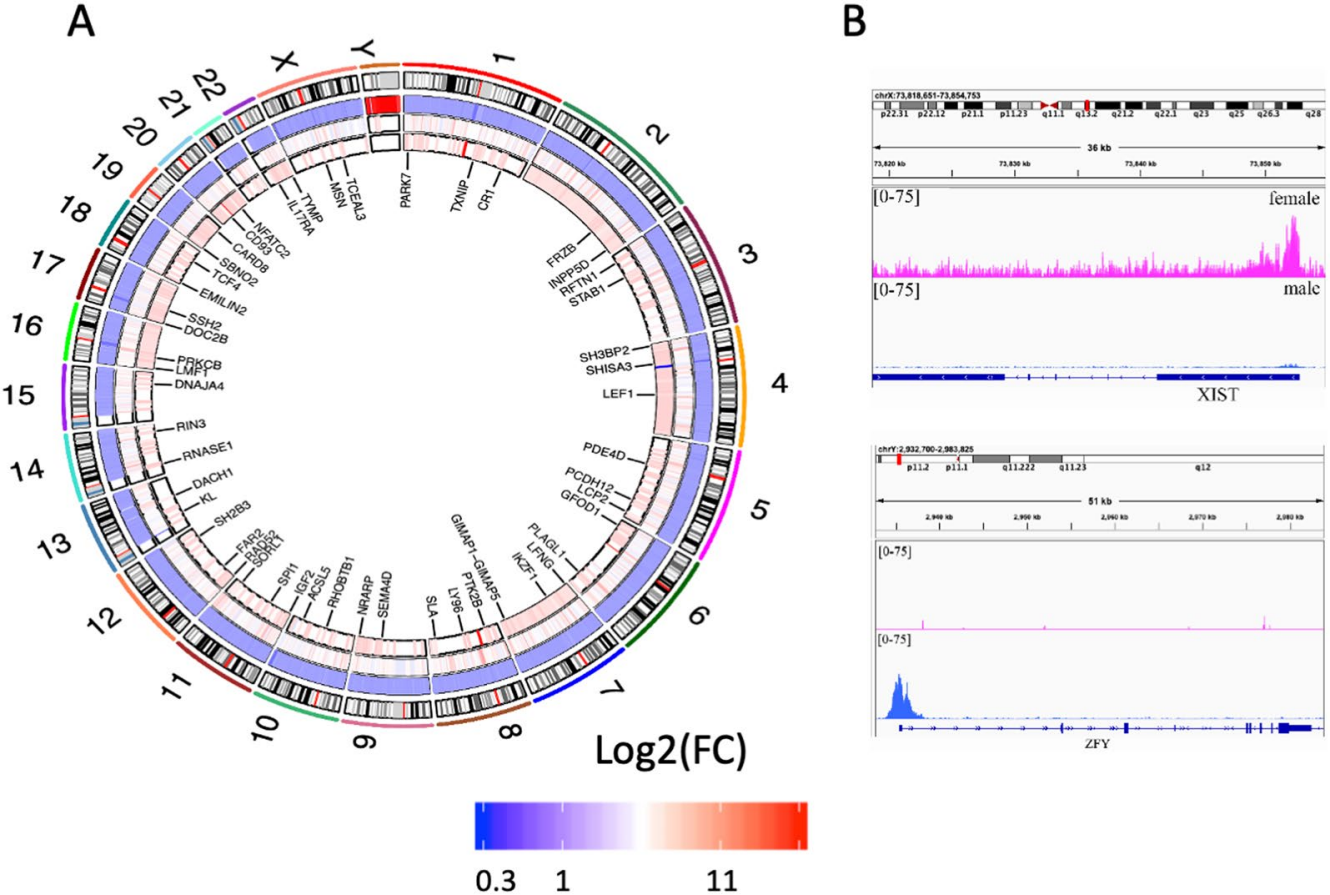
H3K4me3 occupancy is globally affected in the human placenta. (**A**) The Circos plot shows the regions of significantly altered H3K4me3 in humans. The plot indicates the normalized log2 fold-change values for the significant differential regions. The innermost Circos heatmap is for differences in female samples. The outermost ring shows the chromosome location. Circular tracks from outside to inside: heatmap of the differential regions when comparing male and female samples. The next heatmap shows differences in males exposed to DE compared to controls, and the last plot shows the differential regions in samples exposed to DE and control samples. Some genes located in the differential regions are indicated. (**B**) H3K4me3 at XIST (top) was detected only in female placentas, and H3K4me3 at ZFY (bottom) was detected only in male placentas.

To reveal the effects of in utero DE exposure on the transcription regulatory marks at the genome level and to find new targets, we analyzed the genome-wide distribution of H3K4me3 marks using chromatin from the placenta of 4 unexposed and 10 DE-exposed placentas from females and 5 unexposed and 12 DE-exposed placentas from males (the details are provided in the “Methods” section). An overview of the sequencing results is presented in a Circos plot of all the sequencing data (Fig. 4A). We observed a global decrease in H3K4me3 in exposed male samples compared to nonexposed samples and a global increase in H3K4me3 levels in exposed female placentas.

To reveal sex-specific differences in H3K4me3 occupancy, we compared the sequencing data between all samples from females and males (Fig. 4A). The principal component (PCA) analysis showed the substantial difference between male and female placenta samples (Fig. S4A–C).

We also confirmed that H3K4me3 protein level in male placenta was higher by quantitative WB of purified histones from non-exposed male and non-exposed female placenta similar as we did for γH2AX (Fig. S5A). Our analysis showed that H3K4me3 is lower in female compared to male placenta (Fig. S5B).

Our analysis revealed that 817 peaks (FC > 2), which are located near 1201 genes, were differentially expressed between males and females (Fig. 4A, second heatmap in the Circos diagram). The dataset supporting the conclusions of this article is included within the article (Supplementary Dataset File_1). We detected several regions that were unique to either female or male placentas (Table S1). For example, we detected that female placentas had marks in the *XIST* gene, and only male placentas had H3K4me3 marks at the promoter of the *ZFY* gene (Table S1, Fig. 4B). The analysis shows that at the global level, females have lower H3K4me3 occupancy, for example, at the *NANOS3* gene (Fig. S6A).

Next, we asked whether the differences between nonexposed female and nonexposed male H3K4me3 regions were located near genes that share common biological functions. We assigned genes within differential peaks using GREAT and performed functional annotation using the program DAVID. We focused on biological processes analysis. Functional annotation revealed that the differential regions included regions of canonical WNT signaling pathway genes, cell fate commitment, and vasculogenesis genes, among others (Fig. S6B).

**Figure 5:**
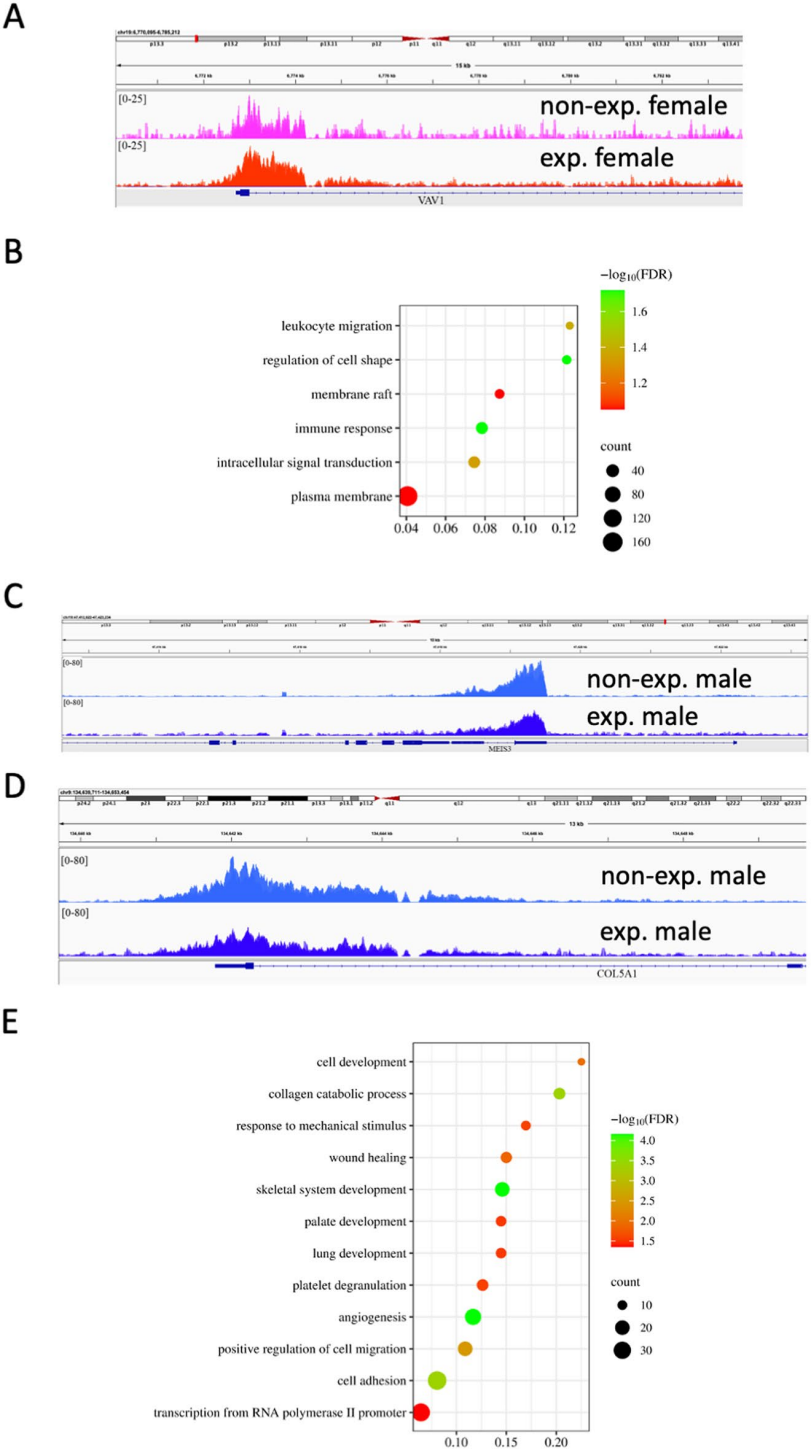
Functional analysis of genes located in altered regions. (**A**) In the *VAV1* gene promoter, there is higher occupancy in H3K4me3 in female placentas exposed to DE. (**B**) Functional annotation of genes located in differential peaks detected in DE-exposed female placentas shows enrichment in biological process terms. (**C**) Histone H3K4me3 occupancy decreased at the promoters of the *MEIS3* and (**D**) *COL5A1* genes. (**E**) Functional annotation of genes located in altered H3K4me3 regions in exposed male placentas.

Next, we asked whether exposure to DE leads to alterations in H3K4me3 occupancies. The differential H3K4me3 were localized in promoters as expected (Fig. S7A,B). Comparative analysis of H3K4me3 between nonexposed and exposed female placentas showed that H3K4me3 occupancy was altered in 466 regions located near 620 genes (FC ≥ 2, FDR ≤ 0.16). The dataset supporting the conclusions of this article is included within the article (Supplementary Dataset File_2). For example, the increased occupancy was determined in the VAV1 gene (Fig. 5A), which encodes a protein that plays an essential role in T-cell and B-cell development and activation. Functional annotation of all genes showed that biological pathways of leukocyte regulation of cell shape and immune response (Fig. 5B) were affected.

In exposed males, we determined that 484 regions were affected, which were located near 597 genes with FC > 2 and FDR < 0.05 (Fig. 4A). The dataset supporting the conclusions of this article is included within the article (Supplementary Dataset File_3). For example, differential regions were localized in the homeobox HOX gene *MEIS1* (Fig. 5C)*,* which plays a crucial role in normal development, and *COL5A1* (Fig. 5D), an important gene for circulatory system development and collagen organization. Functional annotation showed enrichment in skeletal system development, angiogenesis, and collagen catabolic processes, among others (Fig. 5E).

### Altered H3K4me3 regions are enriched in binding sites for NANOG and PRDM6

**Figure 6:**
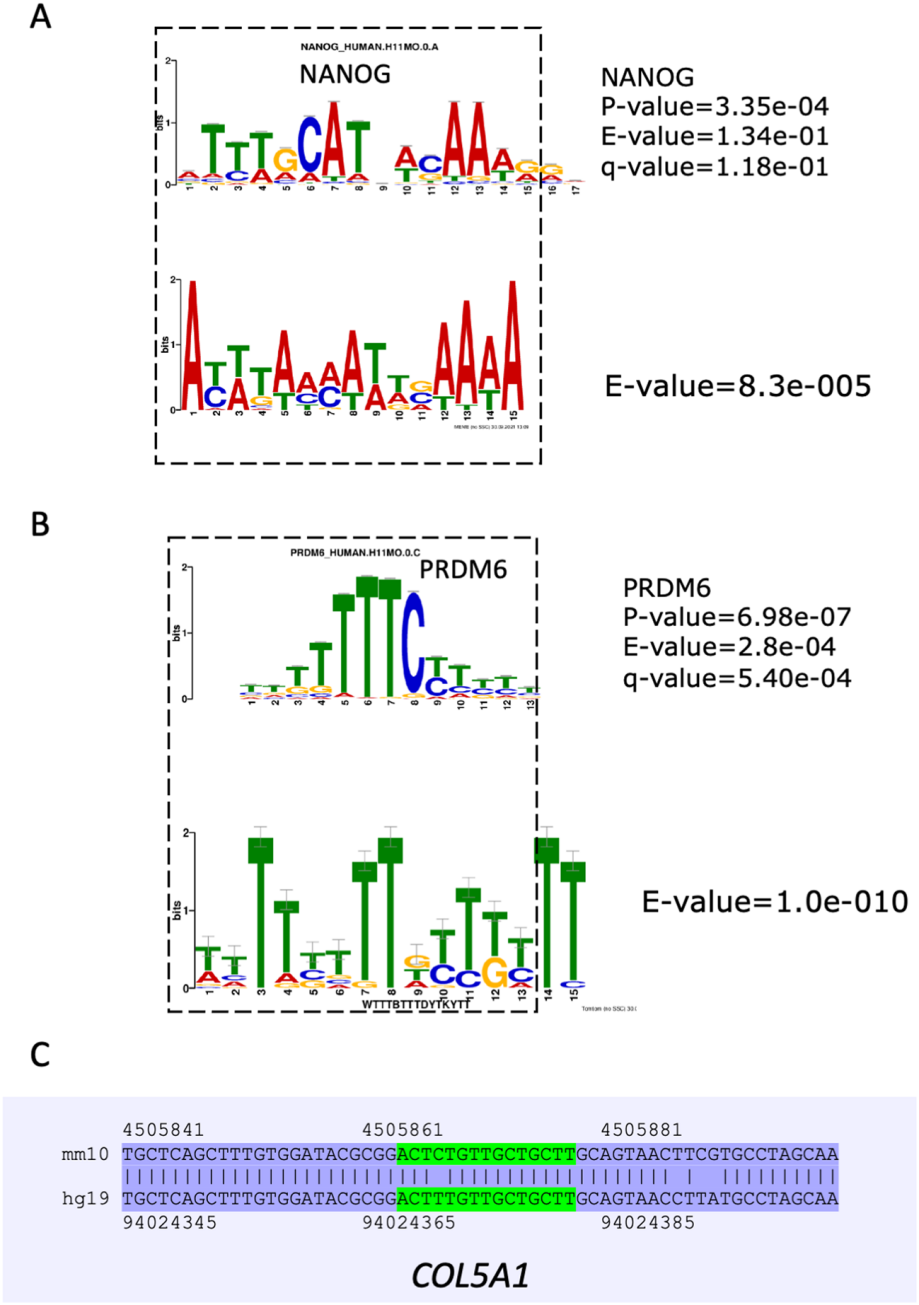
Motif analysis in regions located in differential peaks of female or male placentas. (**A**) An AT-rich motif (bottom) was detected in differential H3K4me3 peaks of DE-exposed female placentas, and part of the motif is similar to NANOG (top). (**B**) A T-rich motif was identified in differential H3K4me3 peaks of DE-exposed male placenta (bottom), and part of the motif is similar to the PRDM6 binding site (top). Dashed squares show the matching part of the motif to the transcription factor binding site. (**C**) The identified PRDM6 binding site was detected in the *COL5A1* gene in DE-exposed male placentas, and the PRDM6 motif is shown in green. The sequence is conserved between mouse and human genomes.

To address whether regulatory DNA motifs are present within the regions containing altered peaks, we used MEME-CHIP^24^. We asked whether there is enrichment for transcription factor-binding sites in DE females and males. We extracted sequences located in differential peaks and performed repeat masking to remove repetitive sequences from the analysis. The resulting files in *fasta* format were processed in MEME-CHIP. The analysis revealed significantly enriched motifs among the differential peaks in female and male placentas exposed to DE. For example, we detected AT-rich motifs in female placentas (e-value = 8.3e−005) (Fig. 6A, bottom). Part of the motif is similar to the NANOG binding site (p-value 3.35e−04, q-value = 1.18e−01) (Fig. 6A, top). Other identified motif was similar to AR, the full list of MEME motifs in Fig. S8.

In male placentas, a T-rich motif was detected (e-value = 1.0e−010), and part of the discovered enriched motif sequence was similar to the PRDM6 binding site (Fig. 6B) (p value 6.98e−07, q-value = 5.40e−04). For example, the T-rich motif was found in the *COL5A5* gene (Fig. 6C). Other identified motifs were similar to SP1/2 and VEZF1 factors, the full list of MEME motifs is in Fig. S9.

### Confirmation of some targets in the mouse placenta

To confirm the sex-specific differences in the placenta, we analyzed identified human targets in the placentas of mice. Mice were dissected at embryonic day 15.5, when the placenta was well formed. The sex of the placenta sample was determined by analysis of the gonads of matching embryos. We fixed the placenta with paraformaldehyde, embedded the tissue in paraffin and analyzed the tissue morphology of H&E-stained sections (Fig. S10). Both male and female placentas had similar morphologies.

**Figure 7:**
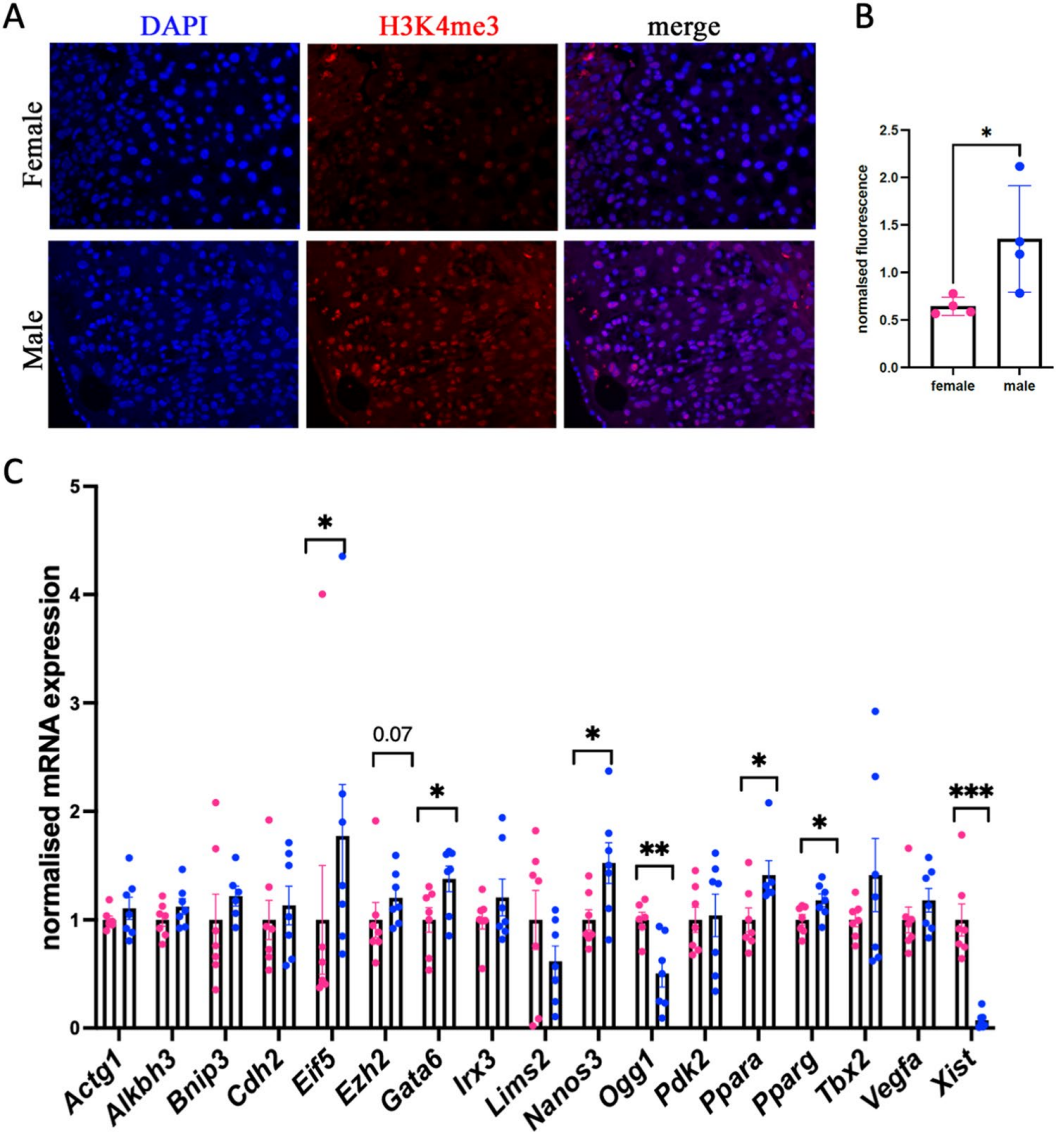
Sex-specific analysis in murine placenta. (**A**) H3K4me3 intensity in the male placenta is higher than that in the female placenta. Female or male placenta sex was identified by dissection of embryos at embryonic day (E15). The placenta was fixed in paraformaldehyde solution, and sections were made. Sections were immunostained with H3K4me3 antibody. Images were taken with the same exposure time, and the fluorescence level from 150 cells was analyzed for each biological replicate using Fiji. (**B**) H3K4me3 fluorescence was normalized to the DAPI signal, and averaged values were plotted and presented as normalized fluorescence. 20 × objective, n = 4 for each group. (**C**) Placenta gene expression shows sexual dimorphism. RNA was extracted from E15 male and female placentas, and cDNA was generated and used for RT‒qPCR. Gene expression was normalized to the housekeeping gene *Rpl37a*, n = 8 for each group, *P < 0.05, **P < 0.01 ***P < 0.001, Mann‒Whitney test.

Since we identified a higher level of H3K4me3 in human placentas in males than in females, we asked whether mice have a similar pattern. To this end, we performed immunofluorescent staining of sections using an antibody against H3K4me3 (Fig. 7A). We performed quantitative analysis of H3K4me3 in a large number of cells. The analysis showed that H3K4me3 was significantly higher in males (Fig. 7B), suggesting the consistency of histone occupancy in mice and humans.

Next, to reveal whether sex-specific changes in histone occupancy are accompanied by gene expression, we performed RT‒qPCR analysis of targets that showed sex-specific differences. We chose important genes encoding protein factors that play essential roles in trophoblast formation (*Gata6*, *Ppara*, *Pparg*, *Tbx2*, *Cdh2*, *Irx3*, *Nanos3*, and *Eif5*) and genes expressed in all cell types, including factors important for the cytoskeleton (*Actg1*), apoptosis (*Bnip3*), DNA repair (*Alkbh3* and *Ogg1*), vasculogenesis (V*egfa* and *Cdh2*) and epigenetics (*Ezh2*). Analysis showed sex-specific differences in genes important for trophoblast function. Gene expression of *Gata6*, *Nanos3*, *Ezh2*, *Ppara*, *Pparg*, and *Eif5* (Fig. 7C) was higher in males, similar to H3K4me3 mark occupancy in humans. We identified that *Xist* mRNA was expressed only in females, similar to the presence of H3K4me3 marks at the promoter of XIST in female human placenta.

Thus, analysis of gene expression in the mouse placenta showed sex-specific alterations in histone H3K4me3 occupancy.

## Discussion

### Effects of DE on telomeres

We observed that exposure to DE leads to alterations in telomere length. Several studies have shown that stress factors lead to alterations in TL. For example, pesticide use for tobacco crops leads to shortened TL Kahl et al.^25^. The use of another pesticide, malathion, also led to a shorter TL (p = 0.03) Andreotti et al.^26^. Oxidative stress could also contribute to telomere damage and shortening^19^. Since we observed an increase in γH2AX and an increase in histone occupancy at 8-oxoguanine glycosylase (*OGG1*), we can imagine a potential role of DE in telomere shortening via oxidation of guanine and global DNA damage.

On the other hand, it is suggested that alterations in the enzyme activity that elongates the telomere repeats (telomerase [*TERT*]) could be relevant to telomere elongation or shortening. The activity of TERT was observed in IUGR placentas, where telomere shortening was explained by decreased *hTERT* mRNA expression^27^. A decrease in TL could also be explained by altered stability at telomeres, which is normally maintained by histone H3K9me3 marks. We observed a decrease in H3K9me3 at telomere regions in response to DE in males. It has been suggested that elevated levels of H3K9me3 at telomeres are required to control the expression of the telomere transcript TERRA; overwise, the increased expression of TERRA leads to telomere shortening^28^. This suggestion was supported by the evidence that the increased H3K9me3 density was detected at longer telomeres^28^. The exact mechanism of this regulation is not well understood.

The decrease in H3K9me3 at telomeres could be due to compromised activities of enzymes introducing H3K9me3, such as *SUV39H1/H2*. For example, exposure to furan, a chemical contaminant that forms in some foods during traditional heat treatment techniques, causes a significant downregulation of *SUV39H1* and *SUV39H2*^29^. Similarly, we suggest that alterations in the activities of these enzymes could be responsible for the lower level of H3K9me3. Further work is required to confirm this connection.

We detected higher levels of H3K9me3 at telomere repeats in males compared to female placentas. This phenomenon could be due to fact that in male placentas the telomeres are longer, so more H3K9me3 could be associated with telomeres.

Thus, our observation suggests that exposure to DE affects H3K9me3 at telomeres, which could be responsible for telomere shortening.

### Effects on the imprinting factors IGF2 and THRA

We identified an increase in H3K4me3 at IGF2 in the female placenta. IGF2 is an imprinted paternally expressed gene, and its function is to regulate the transfer of nutrients from the mother to the fetus in trophoblast cells^30^. The protein encoded by IGF2 plays a role in hormone-dependent pathways in the placenta^31^. Elevated levels of IGF2 were detected in human placentas exposed to ethanol^32^, suggesting that under toxic conditions, the preservation of monoallelic expression is perturbed or the nutrition pathway from mother to child is damaged.

We observed an increase in *THRA* H3K4me3 occupancy in females. Thyroid hormone (TH) is indispensable for normal embryonic and fetal development. Throughout gestation, TH is provided by the mother via the placenta; later in pregnancy, the fetal thyroid gland makes an increasing contribution. An increase in the expression of *THRA* mRNA was observed in the placentas of nutrient-restricted sheep, and it was suggested that this increase in gene expression is an effort to compensate for the lower concentrations of T4 and T3 in maternal blood in nutrient-restricted sheep^33^.

### Global effects on collagen catabolic processes and angiogenesis in males

We identified alterations in many developmental genes, including genes implicated in collagen catabolic processes, in males exposed to DE. The high expression of collagen at the maternal–fetal interface may induce an immune tolerance microenvironment by regulating the differentiation and function of immune cells in the decidua, which is important for successful implantation and pregnancy. A study identified *COL4A1* and *COL4A2* as maternal preeclampsia susceptibility genes^34^. Thus, alterations in histone H3K4me3 at collagen-related genes could reflect perturbations in placental microenvironment processes that may increase the risk of pregnancy complications. Further work is required to determine the role of DE in the cell morphology and shape of placental cell types.

### Potential role of NANOG and PRDM6 in placental pathologies

We observed a large number of binding sites for NANOG in altered H3K4me3 peaks, which is highly expressed in pluripotent stem cells. It has been postulated that NANOG is involved in the pathogenesis of gestational trophoblastic disease, likely through its effect on apoptosis and cell migration^35^. NANOG activation could be implicated in placental growth problems, and its regulation could be disturbed and contribute to placental pathologies.

On the other hand, we found that a large number of peaks have binding sites for PRDM6. This gene encodes a transcriptional repressor gene expressed by vascular precursors^36^. It has been shown that PRDM6 demonstrates transcriptional repressor activity by interacting with class I histone deacetylases and the G9a histone methyltransferase^37^. PRDM6 was found to be significantly associated with systolic blood pressure in individuals of European descent^38^, and *PRDM6* mutations cause common congenital heart defects^39^. Thus, PRDM6 may play an important role in the epigenetic silencing that is crucial for vasculogenesis and heart development; therefore, PRDM6 is a strong candidate as the protein responsible for the observed global decrease in activating histone H3K4me3 in the male placenta in our study.

### Sex-specific differences in vulnerability

The fetus and placenta express paternal antigens. Although recognized by the maternal immune system, changes in both the systemic and local (uterine) immune responses abrogate normal cytotoxic adaptive reactions while enhancing regulatory, tolerogenic responses allowing the pregnancy to progress. Cytokines, growth factors and hormonal signaling pathways play important roles during implantation and parturition. Pregnancy confers unique susceptibility to infection; infectious agents are increasingly implicated in triggering pregnancy complications and infection-induced disruptions in fetal tolerance. We suggest that the observed alterations in immune system genes could lead to a higher susceptibility to infections of male placentas.

Our data confirm the previous observation that male embryos present a greater in utero vulnerability to environmental factors, but the mechanism for the basis of this sex difference is still not clear^40^. There is opinion supported by the work of Cvitic et al.^41^ suggesting that there is less immune compatibility in maternal–fetal interactions for males, which may trigger a higher immune response in male fetuses, thereby resulting in a proinflammatory state.

## Limitations of this study

Human biological samples did not allow us to analyze mRNA expression and proteins encoding transcription factors. Even if our findings remained similar after controlling for some maternal characteristics (age, body mass index, and smoking status), the high variation in the data could be explained by the contribution of other unmeasured factors to which humans are exposed during early life. This study revealed new biomarkers and targets that should be further confirmed and studied using animal or cell culture models to better understand the negative effects of organophosphate metabolites on the human placenta. Another limitation of the present study is related to OP exposure assessment using a single urine sample. OPs have a short biological half-life and are rapidly eliminated in hours to days. Therefore, there is intraindividual day-to-day and within-day variations in OP urinary concentrations. We thus chose to dichotomize exposure variables assuming that groups are less prone to error exposure misclassification and better reflect background exposure levels.

## Conclusions

Our data suggest that exposure to OP metabolites induces sex-specific differences in response to exposure, which could impact fetal growth and development. The identified new factors could be further investigated as biomarkers of exposure to OPs.

## Methods

### The design and setting of the study

All methods were performed in accordance with the relevant guidelines and regulations. In this study, we aimed to analyze the effects of metabolites of organophosphate (OP) pesticides on the human placenta to reveal new biomarkers of exposure to organophosphate pesticides. Ninety-four human placenta samples, 46 males and 48 females, were obtained from the mother–child PELAGIE cohort (see “Methods” section), and the concentrations of DE and DM were measured in maternal urine samples before 19 weeks of gestation (Figs. S11 and S12, respectively). Mitochondrial (mt) DNA and telomere length (TL) copies were analyzed in genomic DNA from placenta samples using the ChIP‒qPCR method. We investigated H3K9me3 occupancy at centromere satellite alfa (SATA) and pericentromeric satellite 2 (SAT2) DNA and at telomere regions. We analyzed histone H3K4me3 occupancies at selected targets in all samples. As targets, we chose genes important for placental development (*PPARA*, *PPARG*, and *KISS1*) and functioning (*IGF2*, *PGR*, *THRA*, and *THRB*), and we studied a marker of oxidative stress (*OGG1*). To reveal new targets of OP that could serve as biomarkers of exposure, we performed genome-wide analysis in a small group of selected samples. In mouse placentas, the morphology, global histone H3K4me3 levels, and gene expression were analyzed at embryonic day 15 (E15).

### The PELAGIE cohort

The PELAGIE (Perturbateurs Endocriniens: Étude Longitudinale sur les Anomalies de la Grossesse, l’Infertilité et l’Enfance) cohort is a mother–child cohort recruited from the general population in Brittany (France) from 2002 to 2006. The pregnant women were included during their first pregnancy visit by gynecologists, obstetricians, or ultrasonographers. One criterion of inclusion was being pregnant from less than 19 weeks of gestation. In the study population, gestational age at inclusion (= at urine sampling) ranged from 6 to 15 weeks of gestation with a median at 11.

First-morning-void urine samples were collected from each pregnant woman only once at their inclusion before 19 weeks of gestation, in which the six nonspecific dialkylphosphate (DAP) metabolites of numerous OP insecticides [diethylphosphate (DEP), diethylthiophosphate (DETP), diethyldithiophosphate (DEDTP), dimethylphosphate (DMP), dimethylthiophosphate (DMTP), and dimethyldithiophosphate (DMDTP)] were measured. The chemical analyses were performed by the LABOCEA Institute (Plouzané, France) with solid-phase extraction (SPE) and liquid chromatography‒electrospray ionization tandem mass spectrometry (LC/MS–MS). Details including quality control/quality assurance procedures and equipment are provided in previous works^42^. At inclusion, pregnant women were asked to complete a questionnaire including demographic, occupational, and medical characteristics, as well as dietary habits and lifestyle. The characteristics of the study population are provided in the table below (Table 3).

**Table 3:** Maternal and child characteristics of the study population (n = 94).

	N (%)	Mean (sd)
Maternal age (years)		30.7 (3.8)
< 25	5 (5.3)	
25–30	35 (37.2)	
30–35	44 (46.8)	
> 35	10 (10.6)	
Maternal pre-pregnancy body mass index (kg/m^2^)		22.4 (3.6)
< 18.5	3 (3.2)	
18.5–25	72 (76.6)	
25–30	14 (14.9)	
> 30	5 (5.3)	
Parity		
Primiparous	35 (37.2)	
Multiparous	59 (62.8)	
Educational level		
Primary school	15 (16.1)	
High school	16 (17.2)	
University	62 (66.7)	
Smoking status at the beginning of pregnancy		
No smoker	80 (85.1)	
Smoker	14 (14.9)	
Parental origin of the mother		
European	93 (98.9)	
Non-European	1 (1.1)	
Gestational age at inclusion (weeks)		10.6 (1.9)
Preterm birth (< 35 weeks)		
No	0 (0.0)	
Yes	94 (100.0)	
Birth weight (g)		3426 (409)
Small for gestational age		
No	88 (93.6)	
Yes	6 (6.4)	

The study population included healthy participants with most of the mothers with a body mass index in the normal range (77%), with a high educational level (68% with a university degree) and few smokers at the beginning of the pregnancy (15%). Ethnicity is a data that cannot be collected in France but we know that only 1 mother has both parents of non-European origin. Similarly, newborns are in good health with no preterm birth and a mean birth weight of 3426 g (6 children were born small for their gestational age). Overall, there is no association between those characteristics and DM or DE levels, except a negative correlation between DM exposure and birthweight (Spearman correlation = − 0.21, p = 0.049) and higher DE levels in women with a university degree (median DE = 0.65 nmol/L) than the other (median DE ≤ LOQ).

At birth, data from maternity records and other biological samples were collected. The samples included a 10 cm^3^ rectangle of placenta, as close as possible to the point of cord implantation, which was then frozen at – 80 °C. At 6 years of age, a neuropsychological examination of the children was organized to study the role of prenatal exposure to potential neurotoxicants, including OPs, on the cognitive performance of the children^42–44^. This examination excluded twins and children with major health issues for which an impact on neuropsychological development is well established (e.g., severe preterm status, specific genetic anomalies). A subgroup of 94 participants of this examination was selected for the present study, including those with the highest participation in the follow-ups of the PELAGIE cohort between birth and 6 years of age and with available placenta samples (approximately 60% of the cohort): 48 girls and 46 boys. For this study, each of these placenta samples was cut at four to six different places, and these pieces were chopped finely to obtain a homogeneous cell composition for each sample. The chopped placenta samples were stored at − 80 °C until use. Urinary metabolite concentrations were converted from micrograms per liter to their molar concentrations (nanomoles per liter). The overall DE concentrations were obtained with the sum of the concentrations of DEP, DETP, and DEDTP (Fig. S11, Table 4). The DM concentrations were obtained as the sum of the concentrations of DMP, DMTP, and DMDTP Fig. S12, Table 4). The limits of quantification (LOQ) for the chemical analyses of maternal urine samples were 1.25, 1.7, 0.02, 0.2, 1, and 0.45 μg/L for DEP, DETP, DEDTP, DMP, DMTP, and DMDTP, respectively. The coefficients of variation at LOQ respectively 19, 19, 20, 17, 19, and 20%.

**Table 4:** Exposure levels expressed in nmol/L (n = 94).

	N (%) > LOQ	Min	Median (Q1; Q3)	Max
DM	75 (81)	< LOQ	24.85 (4.06; 72.78)	895.65
DE	46 (50)	< LOQ	0.054 (< LOQ; 13.26	399.95

*DM* Dimethyl Phosphate metabolites (sum of DMP, DMTP, DMDTP), *DE* Diethyl Phosphate metabolites (sum of DEP, DETP, DEDTP).

### DNA extraction

DNA extraction was performed using DNeasy Blood & Tissue kits (Qiagen) following the manufacturer’s instructions. DNA extraction included an RNAse A treatment step to avoid the presence of RNA. The concentration of DNA was measured using the QuantiFluor dsDNA system (Promega). The quality of DNA was assessed by running samples on a 0.7% agarose gel, where a single band for each sample was observed. The quality of DNA was also confirmed by the ratio of A260 to A280, which was greater than 1.8.

### qPCR analysis of copy number variation (CNV) experiments

The extracted genomic DNA was diluted to obtain a 0.1 ng/μL concentration, and equal amounts of extracted genomic DNA were used for qPCR. Quantitative PCR was performed using a 384-well plate. Each well contained 5 μL of SYBR® Green Master Mix (Bio-Rad), 0.05 μL of each primer pair (100 µM stock solution), 0.9 μL of H2O, and 4 μL of DNA template. qPCR was performed using a CFX 384 Real-Time System (Bio-Rad) with a two-step protocol: initial denaturation at 98 °C for 30 s, followed by a 40-amplification cycle program at 98 °C for 15 s and 65 for 1 min. The melting program was performed after the PCR program. Normalized expression values were calculated with the internal CFX Manager program, provided by 384 CFX Bio-Rad machine using *RLP0* as the reference gene. The primers used in this study are listed in Table S2.

### Histone extraction

Histone extraction was performed using an ab113476-histone extraction kit (Abcam) according to the manufacturer’s instructions. Briefly, equal amounts of placenta (~ 100 mg) were collected from each sample and homogenized using metal beads and TissueLyser II (Qiagen), followed by centrifugation and lysis of the pellet to isolate very basic proteins in the supernatant, such as histones. Protein concentrations were estimated using a Pierce 660 nm Protein Assay (Thermo Scientific).

### Western blotting

Western blots were prepared using the rabbit polyclonal antibody anti-histone γH2AX (Trevigen, 4411-PC-100, 1:1000 dilution). Equal amounts of histone proteins (10 μg) in 10 mM Tris buffer and Laemmli 4X buffer were denatured and run on a 4–15% gradient SDS‒PAGE gel (Mini-PROTEAN® TGXTM Precast Protein Gels). Proteins were transferred onto polyvinylidene difluoride (PVDF) membranes (Millipore, France) using an electroblotter system (TE77X; Hoefer, USA) for 1.15 h. Blocking was conducted using 5% milk in 1X TBS Tween 0.05%. The primary antibody was diluted in 10 mL of the blocking solution, and the membranes were incubated overnight at 4 °C. After three 10-min washes with 1X TBS, each membrane was incubated for 1 h in 40 mL of blocking solution containing the corresponding HRP-conjugated secondary antibodies (GE Healthcare, USA). After another three 10-min washes with 1 × TBS, Western blotting detection reagents (Amersham ECLTM Prime Western blotting Detection Reagents) were used to coat each membrane for the development of the primary and secondary antibody complexes. Specific protein expression for the antibody was then photographed using a molecular imager (ChemiDocTM XRS + System with Image LabTM Software). Ponceau Red-stained H3 histone bands were used to normalize the levels of γH2AX for each sample (Fig. S2). The intensity of the bands was measured in Fiji: ImageJ software.

### Chromatin immunoprecipitation (ChIP)

We performed ChIP using rabbit polyclonal antibodies against H3K4me3 (Millipore, 07-473) or H3K9me3 (Abcam, ab8898). An equal amount of material (~ 50 mg) was weighed from each placenta sample and incubated in 1% paraformaldehyde solution for 10 min to crosslink proteins to DNA. Then, 100 μL of 1.25 M glycine was added to each sample to quench the unbound paraformaldehyde. The samples were centrifuged, and 1 mL of PBS and two metal beads were added to the pellet, which was then homogenized using a TissueLyser (Qiagen). Next, the samples were filtered in a cell strainer, and the resulting solution was pelleted and resuspended in the following buffer: 0.25% (vol/vol) Triton X-100, 10 mM EDTA, 0.5 mM EGTA, and 10 mM Tris, pH 8. Samples were centrifuged at 1100 rpm for 5 min at 4 °C, and the pellet containing cells was resuspended in 300 μL of SDS lysis buffer (1% (wt/vol) SDS, 10 mM EDTA, and 50 mM Tris–HCl, pH 8) supplemented with a protease inhibitor. Chromatin was sonicated in SDS lysis buffer at 60% amplitude for 8 min (20 s on, 20 s off, Qsonica 700 sonicator supplied with a 431C2 cup horn); these parameters allowed us to obtain ~ 300-bp fragments. After sonication, the samples were centrifuged at 12,800 rpm for 10 min at 4 °C, and the supernatant containing sonicated chromatin was transferred and diluted using 1.7 mL of the following buffer: 0.01% (1.1% (vol/vol) Triton X-100, 1.2 mM EDTA, 16.7 mM Tris–HCl, 167 mM NaCl). A solution containing 20 μL of Dynabeads (10002D, Invitrogen) and antibody specific for the histone target was added to the sample tubes and incubated overnight at 4 °C. Before adding the antibody and Dynabeads, 10 μL of each sample was collected as “input samples” (starting material).

After overnight incubation with Dynabeads and the antibody of interest, the beads were washed 5 min each with the following four buffers: (1) low-salt buffer: 0.1% (wt/vol) SDS, 1% (vol/vol) Triton X-100, 2 mM EDTA, 20 mM Tris–HCl, 150 mM NaCl; (2) high-salt buffer: 0.1% (wt/vol) SDS, 1% (vol/vol) Triton X-100, 2 mM EDTA, 20 mM Tris–HCl, pH 8, 500 mM NaCl; (3) LiCl buffer: 0.25 M LiCl, 1% (vol/vol) Igepal, 1 mM EDTA, 10 mM TrisCl, pH 8, 1% (wt/vol) deoxycholic acid; and (4) TE buffer (two washes).

After the washing steps, the beads were treated two times with a 50-μL solution containing 1% (wt/vol) SDS and 0.1 M NaHCO3, pH 9, and were incubated at 65 °C for 15 min to elute the precipitated chromatin from the beads. Subsequently, the eluted chromatin was reverse crosslinked by adding 9 μL of 5 M NaCl and incubating at 65 °C for 4 h. Then, the proteins were removed by adding proteinase K and incubating the samples for 1 h at 45 °C. The precipitated DNA was purified with a MiniElute Reaction Clean-Up kit (Qiagen), and the DNA concentration was measured using a QuantiFluor dsDNA system (Promega). A minimum of ~ 5 ng of DNA was obtained.

### ChIP‒qPCR

Equal amounts of precipitated DNA (0.4 ng) and input samples were used for qPCR analysis (0.1 ng/μL). Quantitative PCR was performed as explained before. Normalized expression values were calculated with the internal CFX Manager program, provided by 384 CFX Bio-Rad machine using the region located far from the promoter as a reference gene. We used the region in *GAPDH* for H3K4me3-ChIP normalization and *RPLP0* for H3K9me3-ChIP. Enrichment of each target in the precipitated DNA was evaluated by calculating the ratio between the average of the normalized ChIP DNA copies and the average of the normalized DNA copies in the inputs.

### ChIP-seq analysis

Quality control of the reads was performed with FastQC v0.11.7. The sequencing read information is presented in Table S2. The number of reads per sample is indicated. Reads were aligned to the human GRCh38 reference using Bowtie (v1.2.3)^45^ (command line parameters: -m1-best-strata-v3), sorted using SAMtools (v1.13)^46^ and directly converted into binary files (BAM). PCR duplicated reads were marked and removed using SAMtools. For the visualization of ChIP-seq tracks, Bedgraph tracks were generated using the GenomeCoverageBed function from BEDtools version v2.27.1^47^. IGV was used to visualize the tracks^48^.

The differential enrichment of H3K4me3 regions in the control and treatment groups was performed using the csaw (v1.26.0) package^49^. First, we counted count reads in the full genome using windowCounts() with spacing = 50, width = 150 and bin = FALSE parameters. Then, normalization factors were calculated using normFactors (). Next, estimateDisp () and glmQLFit () from the edgeR package (v3.34.0)^50^ were used to calculate the log2-fold-change, p value and FDR of the differential regions. The significantly differential regions were selected with FDR < 0.05.

RCircos from R (version_1.2.2) was used to generate a circos heatmap for the normalized log2 fold-change values^51^. The gene function annotation bubble map was generated by a homemade R script with color coding: the darker the color is, the smaller the p value. The size of the dot represents the gene proportion.

### Motif enrichment analysis

The repeat masked sequences of differential H3K4me3 regions were performed by RepeatMasker^52^ version open-4.0.9. Motif identification was performed using MEME-ChIP^24^ with the default parameters. The identified motifs were compared with known motifs using TomTom^53^, and Find Individual Motif Occurrences (FIMO) was used to scan for motif-binding sites^54^.

### Functional annotation analysis

The differential regions were analyzed by GREAT^55^ which assigned the ChIP-seq regions to the genes. The gene list was submitted to DAVID^56,57^. We performed the analysis using ‘functional annotation chart” option of DAVID, where we chose ‘GOTERM_BP_DIRECT” with default parameters.

### Animal experimentation

Outbred Swiss mice (RjOrl:SWISS) were purchased from Janvier lab. Mice were kept in animal facility under standard animal house conditions. After breeding, the day of vaginal plug detection was considered embryonic day 0.5 (E0.5). Pregnant females were dissected at gestational day E15.5 when placenta is well developed. Animals from at least three independent litters were used for all experiments.

### RNA extraction and quantitative PCR

For RNA analysis, placentas from 4 pregnant mice at E15 were dissected. The sex of the placenta was determined by analyzing the gonads of matching embryos. Total RNA was extracted using an RNeasy Plus Mini Kit (Qiagen) according to the manufacturer’s instructions. This kit includes a DNA elimination step. Reverse transcription was performed with 1 µg of RNA using an iScript™ cDNA Synthesis Kit (Bio-Rad). The resulting cDNA was diluted 10 times and used for quantitative RT‒qPCR. The primer sequences used for RT‒qPCR are shown in Supplementary Table S3. RT‒qPCR was performed using iTaq Universal SYBR Green Supermix (Bio-Rad) according to the manufacturer**’**s instructions on a CFX384 Touch Real-Time PCR Detection System (Bio-Rad). The quantification cycle (Cq) values were calculated using the internal CFX Manager program, provided by 384 CFX Bio-Rad machine. The Cq values of *Rpl37a* cDNA were used for normalization. The data were analyzed and are presented as the mean fold change (FC) values compared to the control ± SD, *p < 0.05, **p < 0.01, ***p < 0.001.

### Immunofluorescence on paraffin sections

For immunostaining, the placentas from males or females were fixed in 4% (w/v) PFA solution for 16 h, dehydrated and embedded in paraffin. The sections were deparaffinized and rehydrated, and the epitopes were unmasked in 0.01 M citrate buffer, pH 6, at 80 °C for 45 min. After washing in 1X PBS-0.05% Tween (PBS-T), the sections were incubated with rabbit anti-H3K4me3 antibody (1:500, Merck Millipore, 07-473). The sections with primary H3K4me3 antibody (1:500, Millipore 07-473) were incubated in PBS-T overnight at 4 °C in a humidified chamber. After washing in PBS-T, the sections were incubated with appropriate fluorescent secondary antibodies (1:500) for 1 h in a humidified chamber at room temperature. The sections were counterstained with 0.001% (v/v) 4,6-diamidino-2-phenylindole dihydrochloride (DAPI) and mounted using VECTASHIELD solution. Images were obtained using an AxioImager microscope equipped with an AxioCam MRc5 camera and AxioVision software version 4.8.2 (Zeiss, Germany) with a 20 × objective lens. We analyzed a minimum of 6 images per replicate and quantitated the signal intensity in cells using ImageJ v1.52n. We subtracted the background using regions with no cells. The mean intensity of H3K4me3 was measured, normalized to the signal intensity of DAPI and calculated as the normalized mean fluorescence.

### Statistical analysis

We categorized the urinary concentrations into two groups: nondetected and detected values in urine samples for DE and below and above the median values for DM. The Mann‒Whitney test was used to compare the associations between the two groups. To strengthen our results and control for maternal characteristics that may affect placental function, we additionally performed multivariable linear regression models for the associations observed with the Mann‒Whitney test. We therefore applied multivariable linear regressions considering maternal age, body mass index (> 25 vs. ≤ 25 kg/m^2^), and smoking (yes vs. no) as independent variables in the models.

### Human ethics statement and consent to participate

The research procedures were approved by the Ethics Committee for biomedical studies involving human subjects. Informed consent was obtained from all participants and/or their legal guardian. The adult subjects gave written informed consent, and the 6-year-old children provided verbal and witnessed assent. The French National Commission for the Confidentiality of Computerized ethics committee approved all study procedures (No. 902076).

### Ethics statement using animal

All experimental procedures using animals were authorized by Ministry of National Education and Research of France (Number APAFIS#17473-2018110914399411 v3). The animal facility used for the present study is licensed by the French Ministry of Agriculture (agreement D35-238-19). All experimental procedures followed the ethical principles outlined in the Ministry of Research Guide for Care and Use of the Laboratory Animals and were approved by the local Animal Experimentation Ethics Committee (C2EA-07). All methods were in accordance with ARRIVE guidelines.

## Supplementary Information

Supplementary Information.

## Data Availability

All sequencing and ChIP-seq data from this study are publicly available and have been deposited in the National Center for Biotechnology Information Gene Expression Omnibus. The GEO number is GSE209943.The datasets supporting the conclusions of this article are available in the [GEO] repository, [GSE209943] and hyperlink to datasets https://www.ncbi.nlm.nih.gov/geo/query/acc.cgi?acc=GSE209943.

## Abbreviations

OP: Organophosphate
ACTG1: Actin gamma 1
ALKBH3: AlkB homolog 3, alpha-ketoglutarate dependent dioxygenase
AR: Androgen receptor
BNIP3: BCL2 interacting protein 3
CDH2: Cadherin 2
COL4A1: Collagen type IV alpha 1 chain
COL4A2: Collagen type IV alpha 2 chain
COL5A1: Collagen type V alpha 1 chain
DAP: Dialkyl phosphate
DAVID: The database for annotation, visualization and integrated discovery
DOHAD: Developmental origin of human disease theory
DE: Diethylphosphate
DM: Dimethylphosphate
EIF5: Eukaryotic translation initiation factor 5
EOMES: Eomesodermin
EZH2: Enhancer of zeste 2 polycomb repressive complex 2 subunit
GATA3: GATA binding protein 3
GREAT: Genomic regions enrichment of annotations tool
HBM4EU: Human biomonitoring for European union
IGF2: Insulin like growth factor (IGF2)
*IRX3*: Iroquois homeobox 3
KISS1: KiSS-1 metastasis suppressor
LINE1: Long interspersed nuclear element-1
MEIS1: Meis homeobox 1
MEME: Multiple EM for motif elicitation
NANOG: Nanog homeobox
NANOS3: Nanos C2HC-type zinc finger 3
OGG1: 8-Oxoguanine DNA glycosylase
PPARA: Peroxisome proliferator activated receptor alfa
PPARG: Peroxisome proliferator activated receptor gamma
PRDM6: PR/SET domain 6
SATA: Satelite alfa
SP1: Sp1 transcription factor
SP2: Sp2 transcription factor
SUV39H1: SUV39H1 histone lysine methyltransferase
SUV39H2: SUV39H2 histone lysine methyltransferase
TBX2: T-box transcription factor 2
TEAD4: TEA domain transcription factor 4
TERRA: Telomeric repeat-containing RNA
TERT: Telomerase reverse transcriptase
TERT: Telomerase reverse transcriptase
THRA: Thyroid hormone receptors alpha
TNF: Tumor necrosis factor
VEGF: Vascular endothelial growth factor A
VEGFA: Vascular endothelial growth factor A
VEZV1: Vascular endothelial zinc finger 1
XIST: X inactive specific transcript
ZFY: Zinc finger protein Y-linked
