# Supplementary material for: Genome-wide analysis of sex-specific differences in the mother–child PELAGIE cohort exposed to organophosphate metabolites
Source: Sci Rep. 2023 May 17;13:8003. doi: 10.1038/s41598-023-35113-8 (PMC10192208; doi:10.1038/s41598-023-35113-8)
Supplement: Supplementary file 1 — Supplementary Information. [file 41598_2023_35113_MOESM1_ESM.pdf]

Martina Capriati[1]*, Chunxiang Hao[2]*, Shereen Cynthia D'Cruz[1]*, Christine Monfort[1], Cecile Chevrier[1], Charline Warembourg[1], Fatima Smagulova[1]#

Genome-wide analysis of sex-specific differences in the mother-child PELAGIE cohort exposed to organophosphate metabolites

[1] Univ. Rennes, EHESP, Inserm, Irset (Institut de recherche en santé, environnement et travail) - UMR_S 1085, F-35000, Rennes, France
[2] School of Medicine, Linyi University, Linyi 276000, China

*These authors have contributed equally to this work
# Correspondence: fatima.smagulova@inserm.fr

Supplementary Information

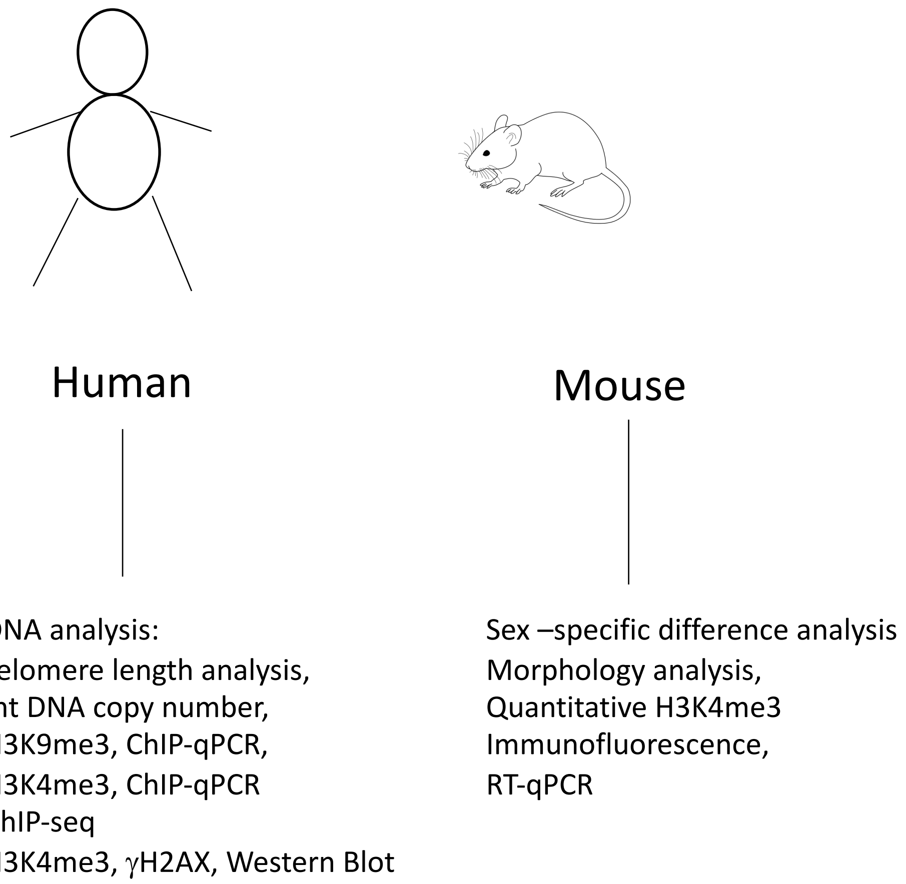

Figure S1. Experiments performed in human and mouse placentas.
Mouse image was taken from Wikimedia Commons
(https://commons.wikimedia.org/wiki/Main_Page).
The picture was generated in PowerPoint (Microsoft office).

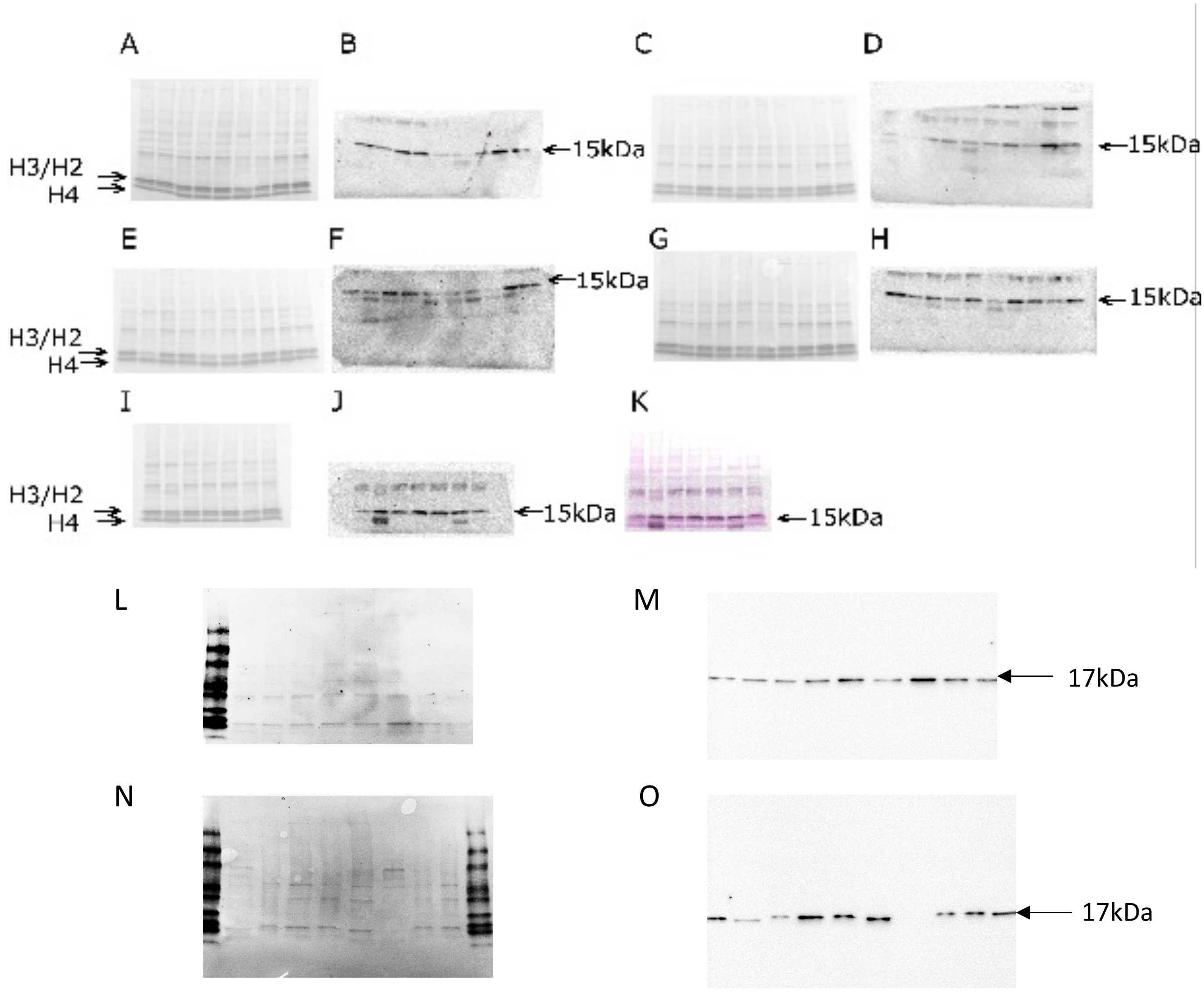

Figure S2. The WB images, Ponceau Red and γH2AX, (A,B) samples 1-9, (C,D), samples 10-19, (E,F), samples 20-29, (G,H), samples 30-39, (I,J), samples 40-46, (K) is a merged image of Ponceau Red and γH2AX of samples 40-46, (L,M) Ponceau Red and H3K4me3 blots of non-exposed female placentas, (N,O) Ponceau Red and H3K4me3 blots of non-exposed male placentas.

A

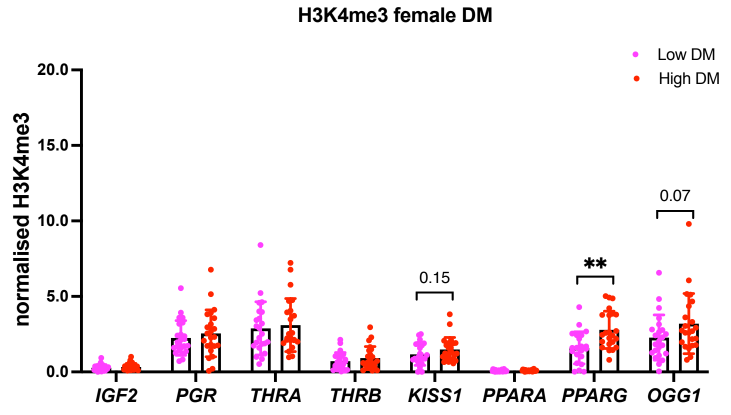

B

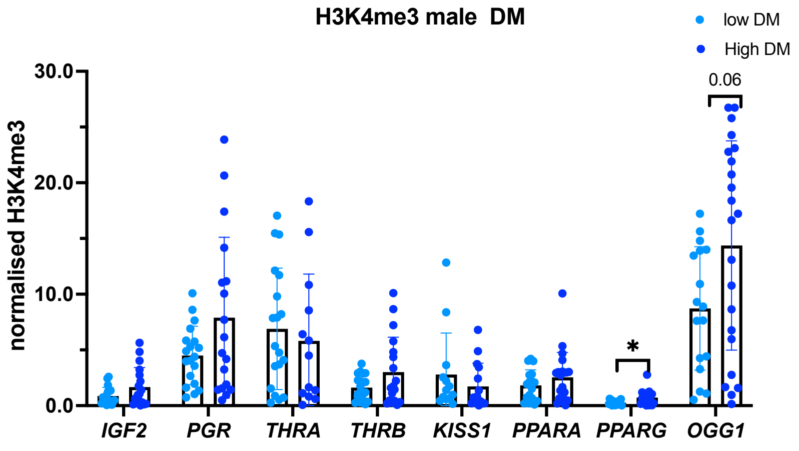

Figure S3. Histone H3K4me3 occupancy at developmental genes in placentas. The effects of DM on female (A) or (B) male placentas, *p<0.05, **p<0.01, Mann–Whitney test.

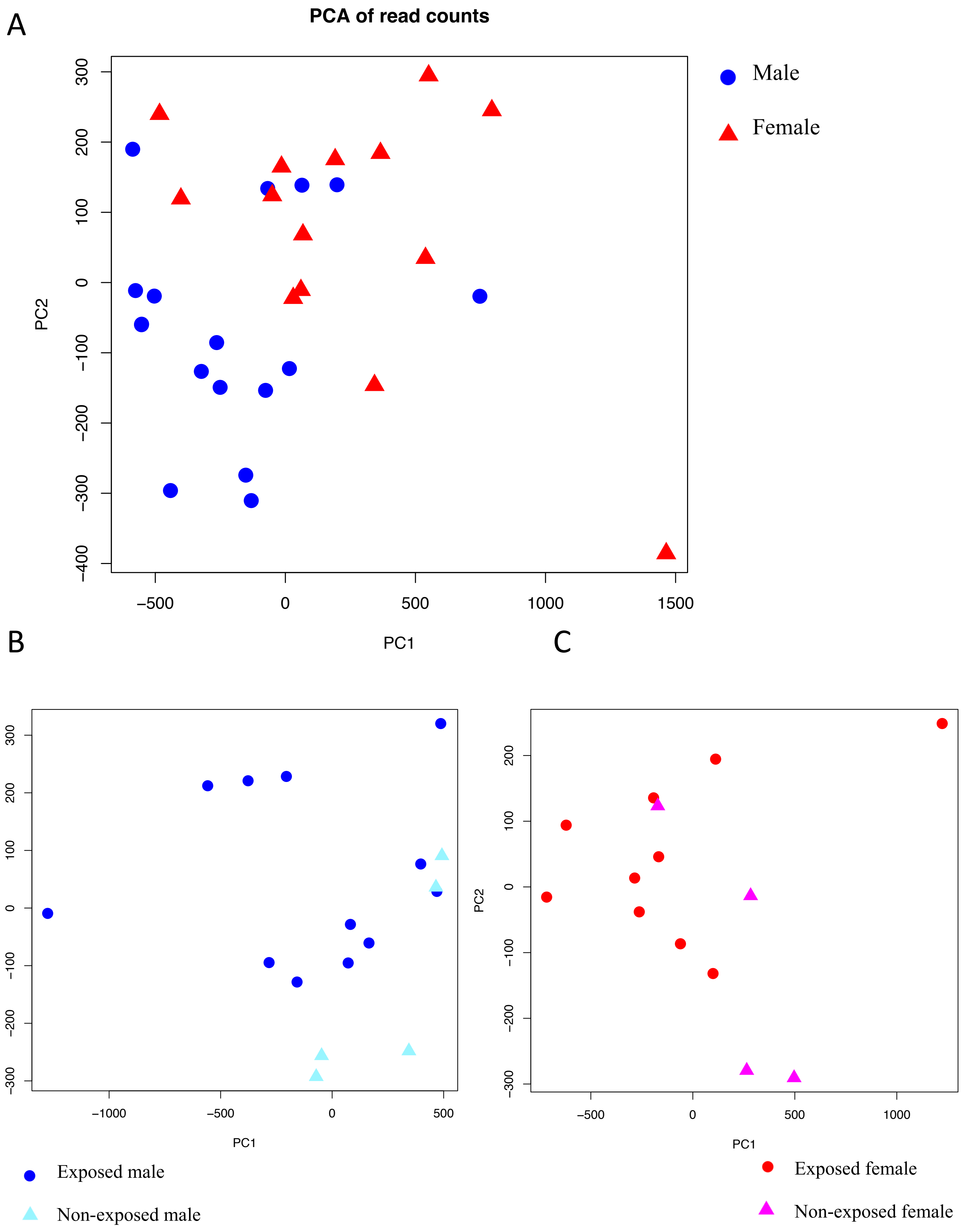

Figure S4. PCA analysis of sequencing data. A) male versus female B) non-exposed male versus exposed male placenta. C) non-exposed female versus exposed female placenta.

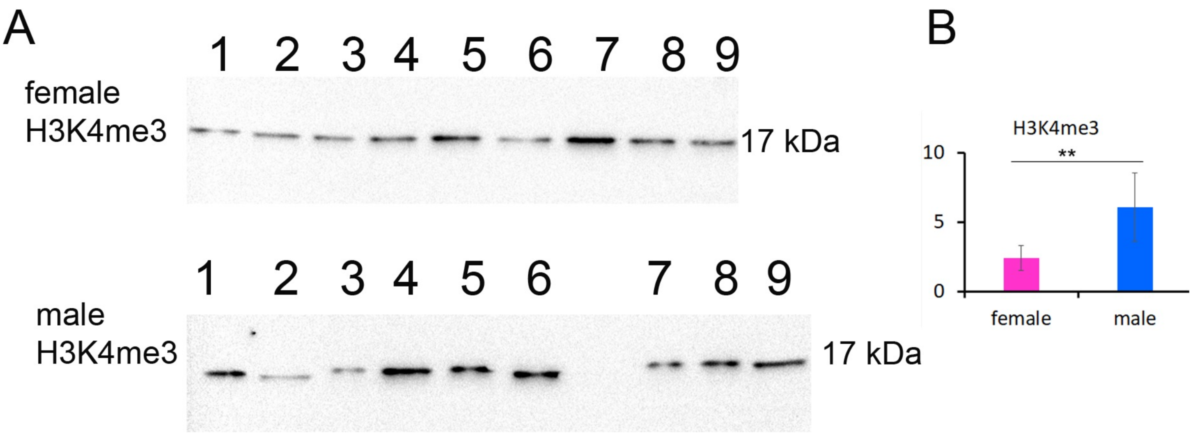

Figure S5. Quantitative analysis of H3K4me3 in unexposed placenta, A) H3K4me3 in non-exposed female (top) and non-exposed male (bottom). B) Quantitative analysis of H3K4me3 WB signal. The histones were extracted as described in Methods section and 5 ug was loaded on the gel. The proteins were transferred on membrane and blotted against rabbit H3K4me3 (1:10000, Merk Millipore, 07-473). H3K4me3 signal was normalised to Ponceau red major band, the data were averaged, plotted and presented as normalised values. The statistical significance was assessed by Mann-Whitney test, *p<0.05. Each lane represents a different placenta sample.

A

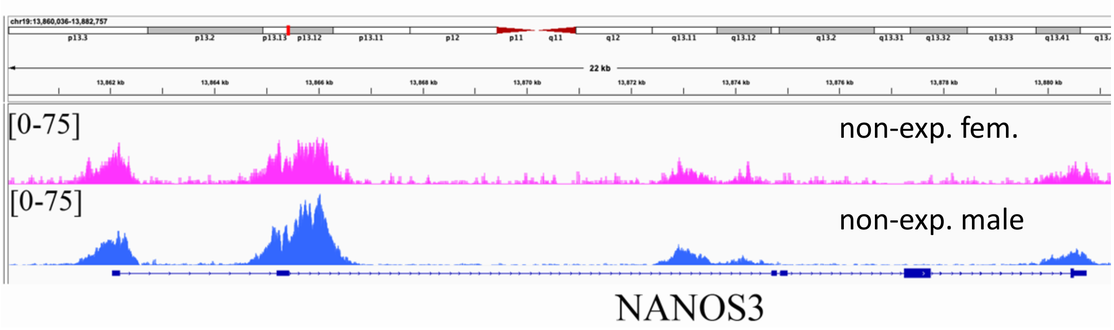

B

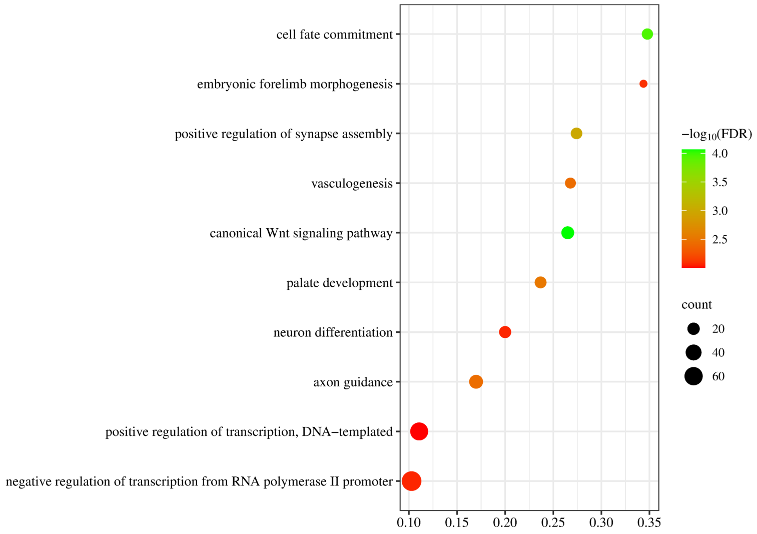

Figure S6. Histone H3K4me3 occupancy has a sex-specific pattern. A) H3K4me3 occupancy is higher in *NANOS3* in human male placenta. B) Functional annotation of genes located in differential regions between non-exposed male and non-exposed female placentas.

A

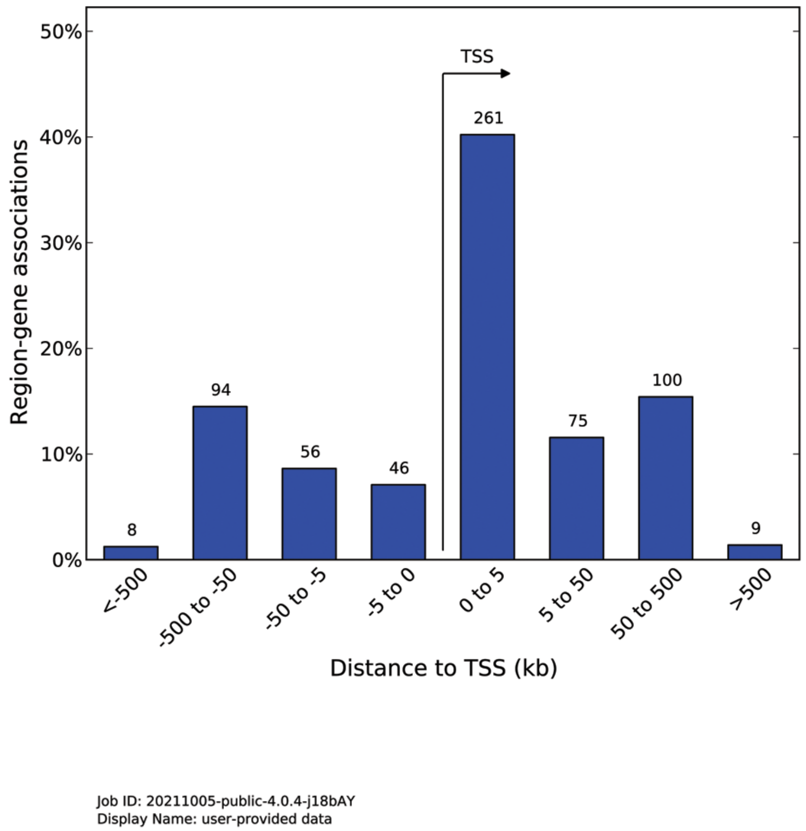

B

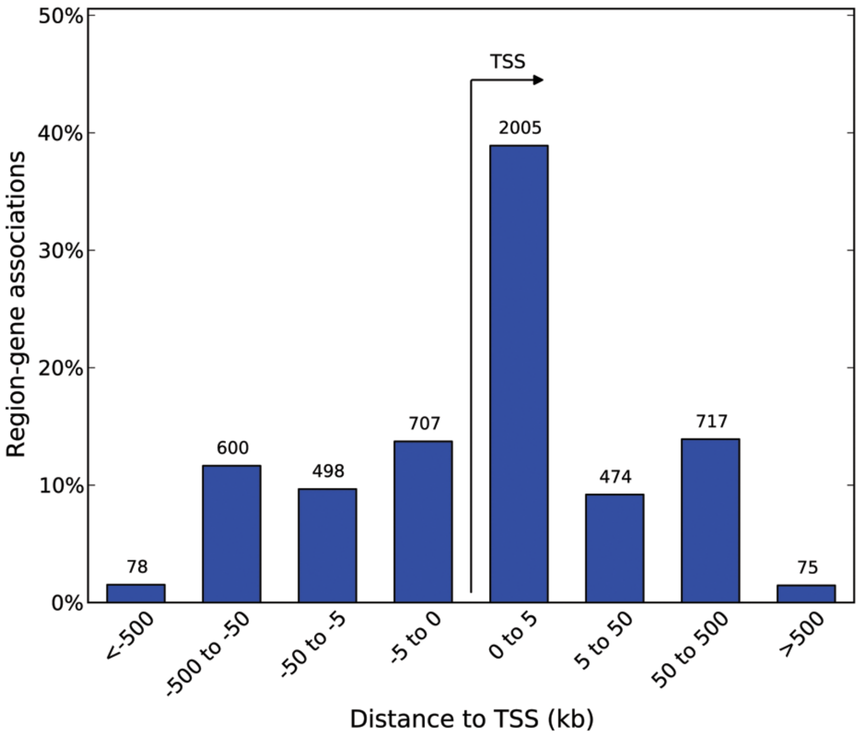

Figure S7. Distance to TSS in differential H3K4me3 peaks identified in DE-exposed (A) female and (B) male placentas. The distance was generated by GREAT

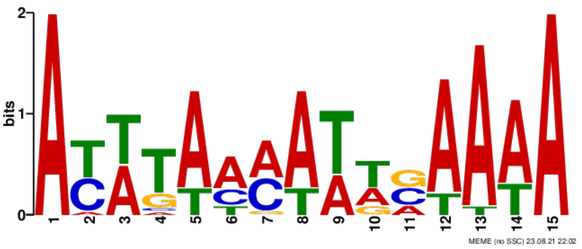

Match, p-value

HXC10 (HOXC10), 1.27e-04
NANOG, 5.57e-04
FOXJ3, 7.16e-04

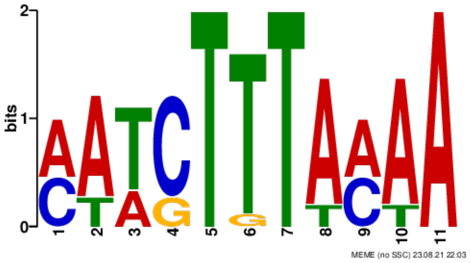

none

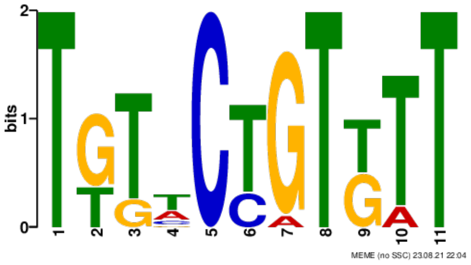

ANDR (AR), 1.98e-05
BPTF, 4.67e-04

Figure S8. The motifs identified by MEME in differential H3K4me3 peaks in female placentas

Match, p-value

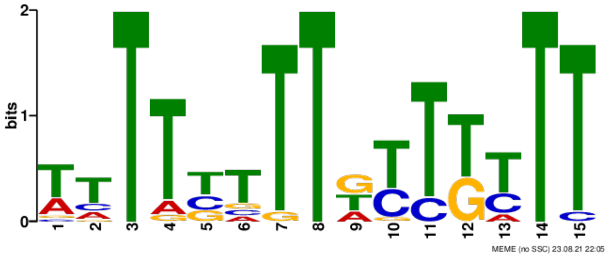

PRDM6, 1.09e-06
CPEB1, 9.71e-07
ANDR (AR), 6.57e-06
FOXJ3, 1.24e-05
CDX2, 5.87e-05

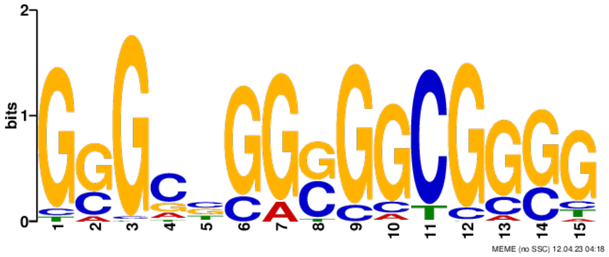

SP1, 2.4e-06
SP2, 1.15e-07
ZNF148, 1.05e-06
TBX15, 3.10e-06

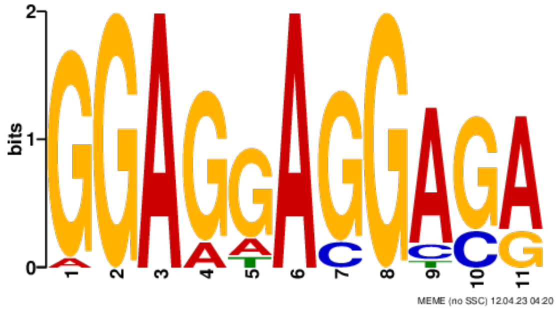

VEZF1, 2.95e-05
WT1, 9.81e-05
ZNF263, 1.17e-04

Figure S9. The motifs identified by MEME in differential H3K4me3 peaks in male placentas

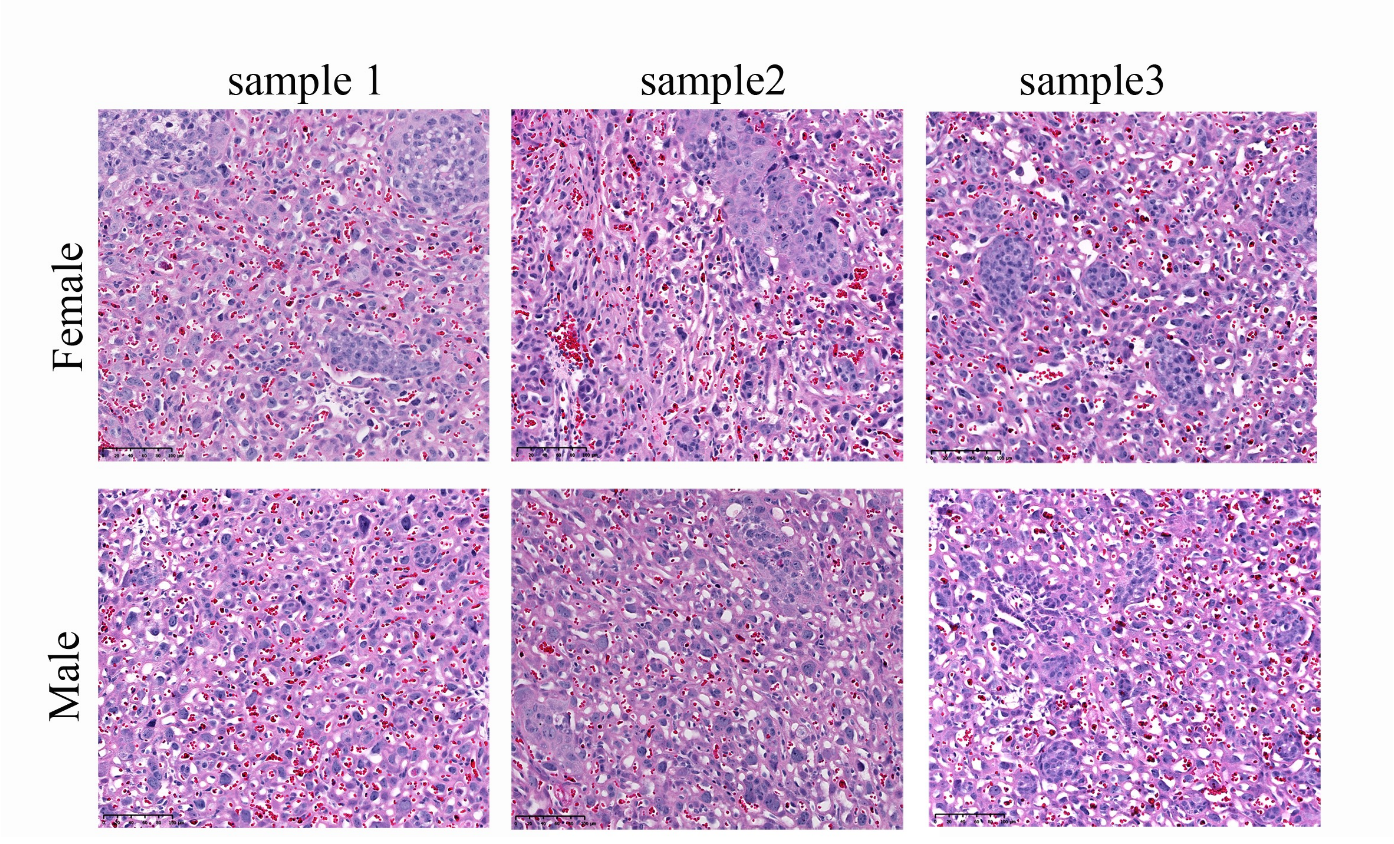

Figure S10. Morphology of murine placenta. Placentas from E15 pregnant Swiss female mice were dissected, and the sex was determined by analysis of matching embryos. The placenta was fixed in paraformaldehyde, embedded in paraffin block and stained with hematoxylin & eosin. There are no apparent morphological differences in male and female placentas, 20× objective, bar is 250 nm.

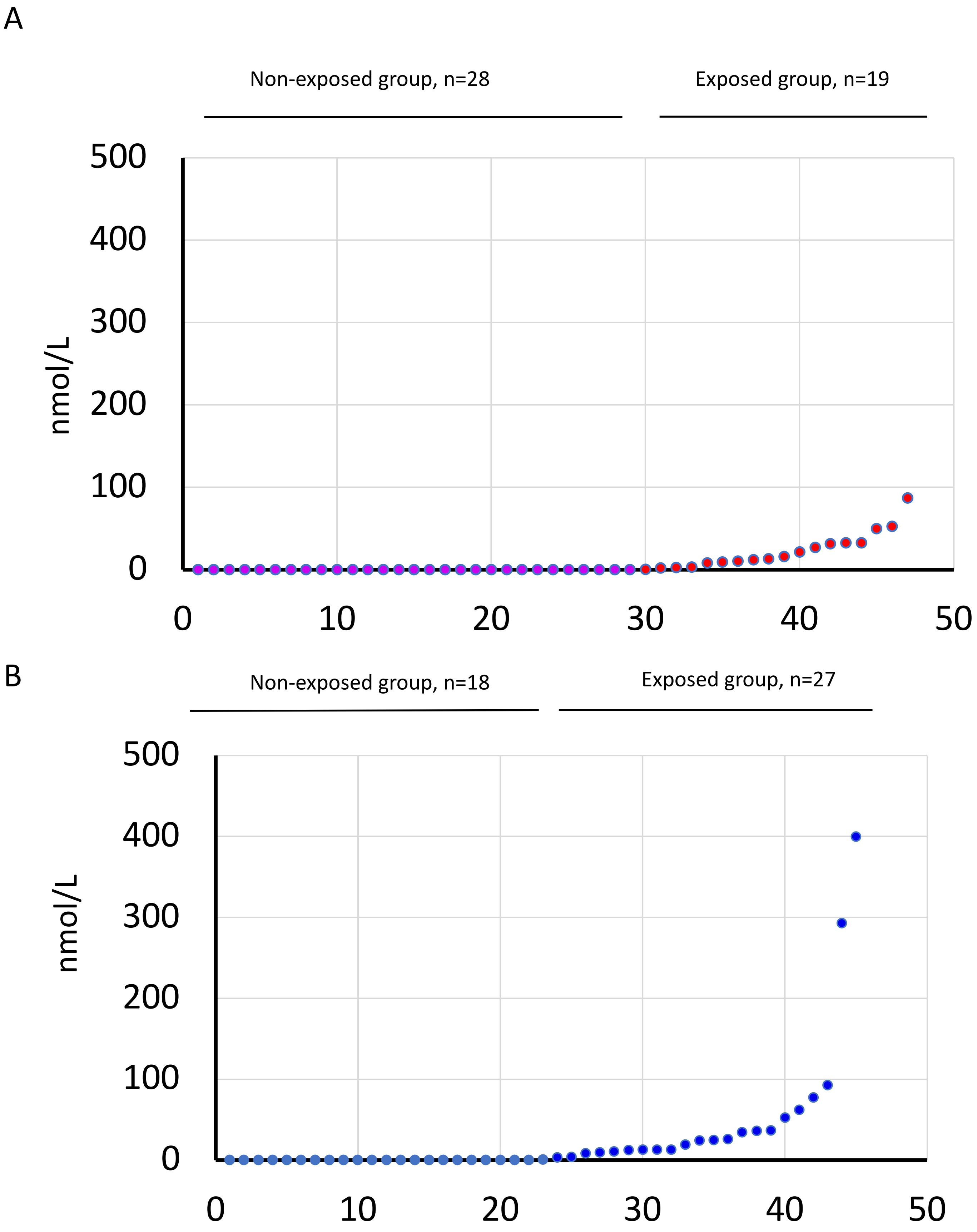

Figure S11. Concentration of DE in urine samples of pregnant woman carrying A) female or B) male babies. The chemical analyses were performed with solid-phase extraction (SPE) and liquid chromatography–electrospray ionization tandem mass spectrometry (LC/MS-MS).

A

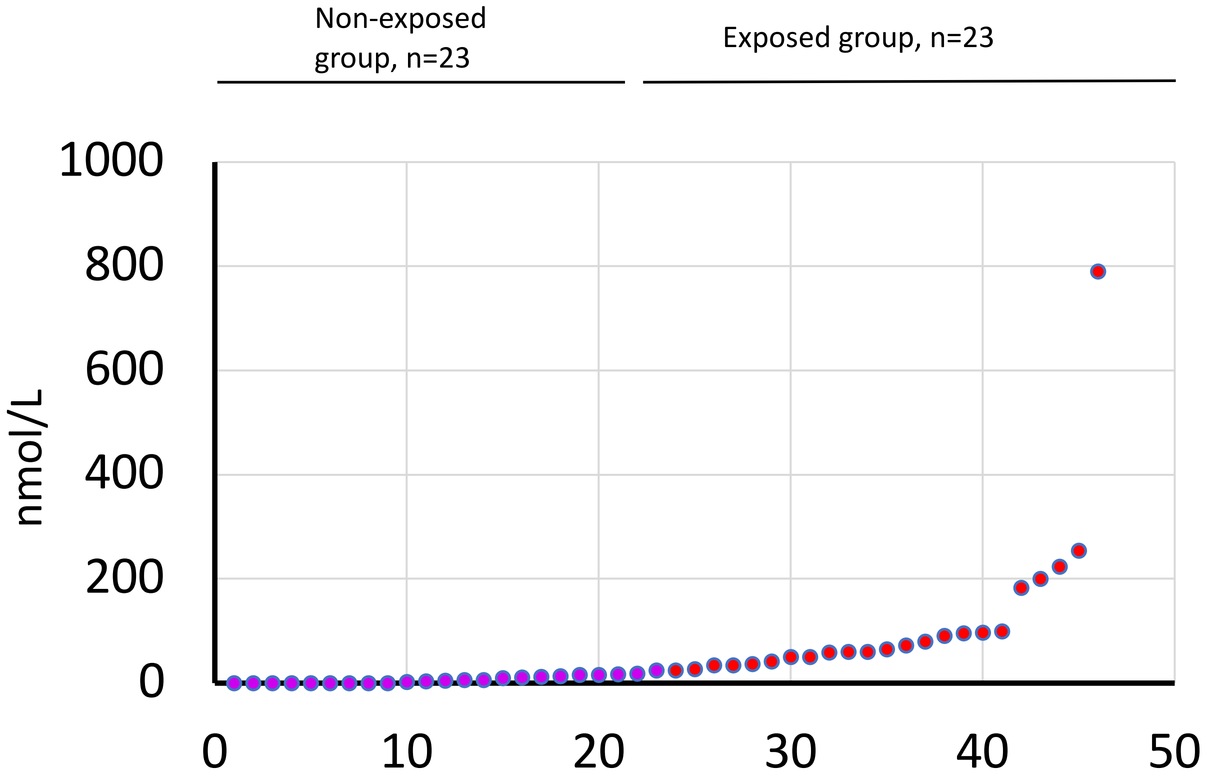

B

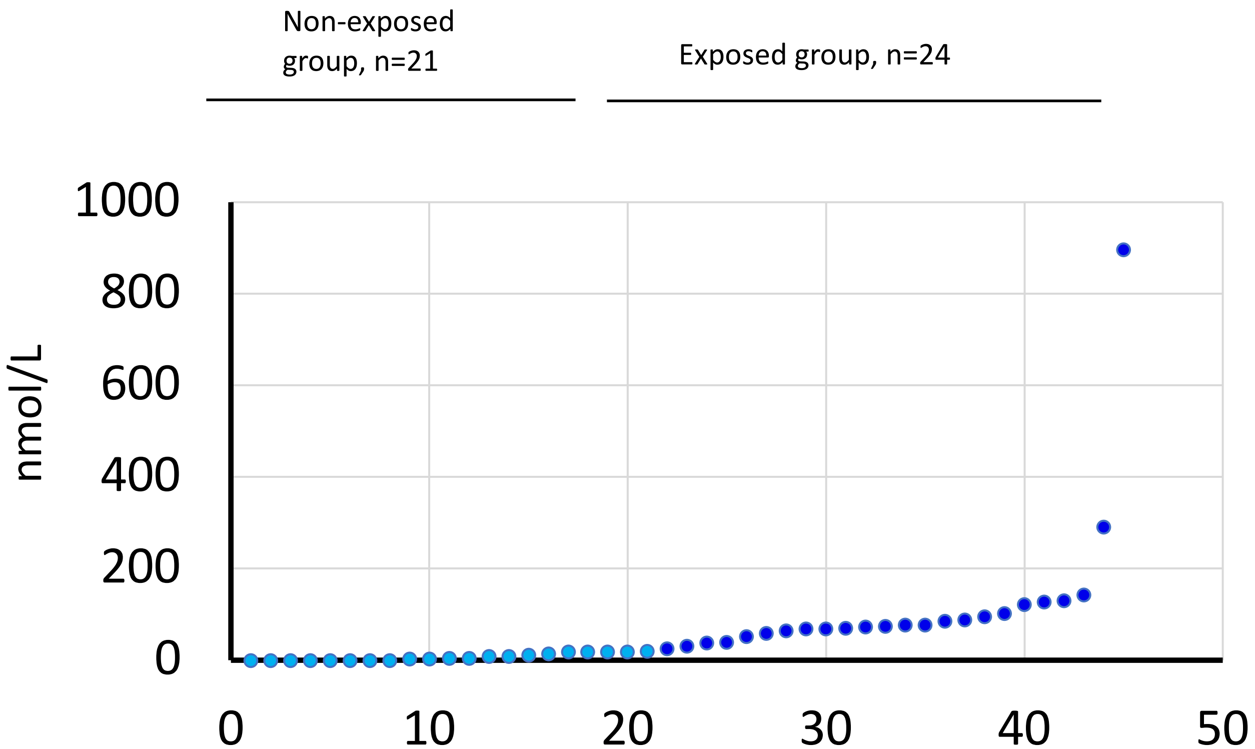

Figure S12. Concentration of DM in urine samples of pregnant women carrying A) female or B) male babies. The chemical analyses were performed with solid-phase extraction (SPE) and liquid chromatography–electrospray ionization tandem mass spectrometry (LC/MS-MS).

Table S1. Sex-specifique unique H3K4me3 peaks

Chr	start	end	FC.		
chr21	10325201	10326500	36,2	BAGE2	female
chr22	18730501	18730800	17,2	FAM230E	female
chrX	73851701	73852550	19,5	XIST	female
chrY	13478001	13480150	3516	UTY	male
chrY	2934701	2936600	3102,8	ZFY	male
chrY	7273151	7275000	3089,9	PRKY	male
chrY	12904551	12906400	2958,1	DDX3Y	male
chrY	2840851	2842300	2783,1	RPS4Y1	male
chrY	12662001	12663600	2780,6	TTTY14	male
chrY	19076051	19077750	2606,3	TTTY14	male
chrY	20575451	20576600	2580	EIF1AY	male
chrY	19744151	19745350	2115,7	KDM5D	male
chrY	13703751	13704750	2052,7	TMSB4Y	male
chrY	3003101	3003900	1882,6	LINC00278	male
chrY	19567201	19567900	1781,8	TXLNGY	male
chrY	13751501	13752150	1776,3	TMSB4Y	male
chrY	12420901	12421800	1739,9	GYG2P1	male
chrY	13435851	13436550	1728,9	UTY	male
chrY	6910601	6911500	1714,9	TBL1Y	male
chrY	56727551	56728750	907,6	SPRY3	male
chrY	56762901	56764150	876,4	SPRY3	male
chrY	56734151	56735400	663	SPRY3	male
chrY	11214501	11215100	26	XR_002958840.1	male

Table S2. Sequencing reads in ChIP-seq data.

DE	Method	sample	Total reads	Uniquely mapped
0,00	ChIP-seq	FMSV210	58469870	49917431
0,00	ChIP-seq	FMSV211	56809638	50036591
0,00	ChIP-seq	FMSV212	38202685	33472325
0,00	ChIP-seq	FMSV213	63830691	55149337
0,00	ChIP-seq	FMSV214	41903085	36891551
4,15	ChIP-seq	FMSV199	50703707	43156829
10,73	ChIP-seq	FMSV200	52471756	44385017
0,29	ChIP-seq	FMSV201	46457702	39298596
25,25	ChIP-seq	FMSV202	43401029	37367728
34,68	ChIP-seq	FMSV203	70417714	60156842
399,95	ChIP-seq	FMSV204	43981834	37356384
0,38	ChIP-seq	FMSV205	39119780	34185634
0,11	ChIP-seq	FMSV206	51128015	44436324
77,76	ChIP-seq	FMSV207	50787916	44052000
19,36	ChIP-seq	FMSV208	45946674	39286067
0,36	ChIP-seq	FMSV209	26677260	22884584
62,26	ChIP-seq	FMSV215	40732171	35492160
ND	Input	FMSV216	56057772	46734881
average			48727739	41903349

DE	Method	sample	Total reads	Uniquely mapped
0,00	ChIP-seq	FMSV264	63729585	53597010
0,00	ChIP-seq	FMSV270	41156918	34992373
0,00	ChIP-seq	FMSV274	51216463	43758973
0,00	ChIP-seq	FMSV275	57496284	48930610
0,00	ChIP-seq	FMSV276	49317295	42058653
21,54	ChIP-seq	FMSV265	28708531	24570323
32,48	ChIP-seq	FMSV266	109690028	92838163
0.2	ChIP-seq	FMSV267	37,228,162	31,835,239
0,21	ChIP-seq	FMSV268	50635043	43120161
86,95	ChIP-seq	FMSV269	51978253	45160470
32,80	ChIP-seq	FMSV271	61507753	52722748
16,18	ChIP-seq	FMSV272	48604509	41870839
0.2	ChIP-seq	FMSV273	44,573,181	38,641,520
3,34	ChIP-seq	FMSV278	48696930	42120559
average			55228132,7	47145073,5

Table S3. Primers used in this work.

gene/region	forward	reverse	target	description
Actg1	CTGGCACCACACCTTCTACA	GGAACAGAACCCTGCGTCAT	mouse RNA	actin, gamma, cytoplasmic 1
Alkbh3	TCTGTAGAAGTGTCACCGAGGG	GAGTGCCCAAGGAATACCACTC	mouse RNA	alkB homolog 3, alpha-ketoglutarate-dependent dioxygenase
Bnip3	GTAGAACTGCACTTCAGCAA	AAGCTGTGGCTGTCTATTTC	mouse RNA	BCL2/adenovirus E1B interacting protein 3
Cdh2	TGGACATGTTGTTAGGGCGG	CCGGCACCATCAGTATGAGC	mouse RNA	cadherin 2
Eif5	TGTGTAATGACAGCAGGAAA	TCGGTACCCACAGGCTTTAC	mouse RNA	eukaryotic translation initiation factor 5
Ezh2	CAAAGGATACAGACAGTGACAGAG	CCGAGAATTTGCTTCAGAGGAG	mouse RNA	enhancer of zeste 2 polycomb repressive complex 2 subunit
Gata6	GCTCACATCTTCTCTGGGTGG	GGCCATTCATCTTGCTGTAGAGAC	mouse RNA	GATA binding protein 6
Irx3	CGCCTCAAGAAGGAGAACAAGA	CGTCGCTCCATAAGCAT	mouse RNA	Iroquois related homeobox 3
Lims2	TGACCTGGGCTTTGTAAAGA	GTCAGCTCCTTCCACAGTTG	mouse RNA	LIM and senescent cell antigen like domains 2
Nanos3	CGGCCTGACAAGGCAAAGAC	CACCATGGTCCTCCCCACTC	mouse RNA	nanos C2HC-type zinc finger 3
Ogg1	GGCCCAGAAATTCCAAGGTGTGA	AGGTCCAAAGGCCTGGCAGA	mouse RNA	8-oxoguanine DNA-glycosylase 1
Pdk2	GAAGACGTCATTCACTTTCC	TTAGAGTCCGGTGGTCCT	mouse RNA	pyruvate dehydrogenase kinase, isoenzyme 2
Ppara	GAGTGCAGCCTCAGCCAAGTT	GCAGGCCACAGAGCGCTAA	mouse RNA	peroxisome proliferator activated receptor alpha
Pparg	TCCAGCATTTCTGCTCCACAC	TTGATCGCACTTTGGTATTCTTGG	mouse RNA	peroxisome proliferator activated receptor gamma
Tbx2	CTGACAAGCATGGCTTTACCA	CGGAAGGTGCTGTATGGGAG	mouse RNA	T-box 2
Vegfa	GGCTTTGTTCTGTCTTTCTTTGGT	TCATGCGGATCAAACCTCACC	mouse RNA	vascular endothelial growth factor A
Xist	TAAACCATGAAGCCTACTGTAACTTCC	CATCCAGCACTTCCCTATGTCTA	mouse RNA	inactive X specific transcripts
Rpl37a	TGGGGCCTGGACCTACAA	GCAGGGCTTCTACTGGTCTT	mouse RNA	ribosomal protein L37a
h_chr1_SAT2*	CATCGAATGGAAATGAAAGGAGTC	ACCATTGGATGATTGCAGTCAA	human H3K9me3 ChIP	Satellite II *
h_chr4_SATA*	CTGCACTACCTGAAGAGGAC	GATGGTTCAACACTCTTACA	human H3K9me3 ChIP	Satellite alfa *
h_chr11_IGF2	TCGTCTGATTGTCCAGGGAGGA	CTGACCTCATTTCCCGATACCTTT	human H3K4me3 ChIP	insulin like growth factor 2
h_chr3_OGG1	CCTACACCTCAGGAAAGCCG	GTGCTGTTTAACAACCTTCCCTG	human H3K4me3 ChIP	8-oxoguanine DNA glycosylase
h_chr17_THRA	TGCAAATGCCTTGCGATTGA	CCCAGCGACCTCCTAGCAA	human H3K4me3 ChIP	thyroid hormone receptor alpha
h_chr3_THRB	ACCCCGAGTCCTACGCAC	GTAATGACGCCCAAACCCG	human H3K4me3 ChIP	thyroid hormone receptor beta
h_chr1_KISS1	GGAGCCTCTGAGGTGACGA	TAAAAGGGATGTGATCAGGGAGC	human H3K4me3 ChIP	KiSS-1 metastasis suppressor
h_chr12_GAPDH	GCTCACTGGCATGGCCTTCCGTGT	TGGAGGAGTGGGTGTCGCTGTTGA	human H3K4me3 ChIP	glyceraldehyde-3-phosphate dehydrogenase
TEL	CGGTTTGTTTGGGTTTGGGTTTGGGTT TGGGTTTGGGTT	GGCTTCCCTTACCCTTACCCTTACCCTTACCCTTA CCCT	human CNV	telomeres DNA
RPLP0/36B4	CAGCAAGTGGGAAGGTGTAATCC	CCCATTCTATCATCAACGGGTACAA	human CNV	ribosomal protein lateral stalk subunit P0
mt DNA	CACCAGCCTAACCAGATTTC	GGGTTGTATTGATGAGATTAGT	human CNV	mitochondrial DNA

* Alexiadis V, Ballestas ME, Sanchez C, Winokur S, Vedanarayanan V, Warren M, et al. RNAPol-ChIP analysis of transcription from FSHD-linked tandem repeats and satellite DNA. Biochim Biophys Acta. 2007;1769:29–40.
